# Supplementary figures and images for: Natural CMT2 Variation Is Associated With Genome-Wide Methylation Changes and Temperature Seasonality
Source: PLoS Genet. 2014 Dec 11;10(12):e1004842. doi: 10.1371/journal.pgen.1004842 (PMC4263395; doi:10.1371/journal.pgen.1004842)

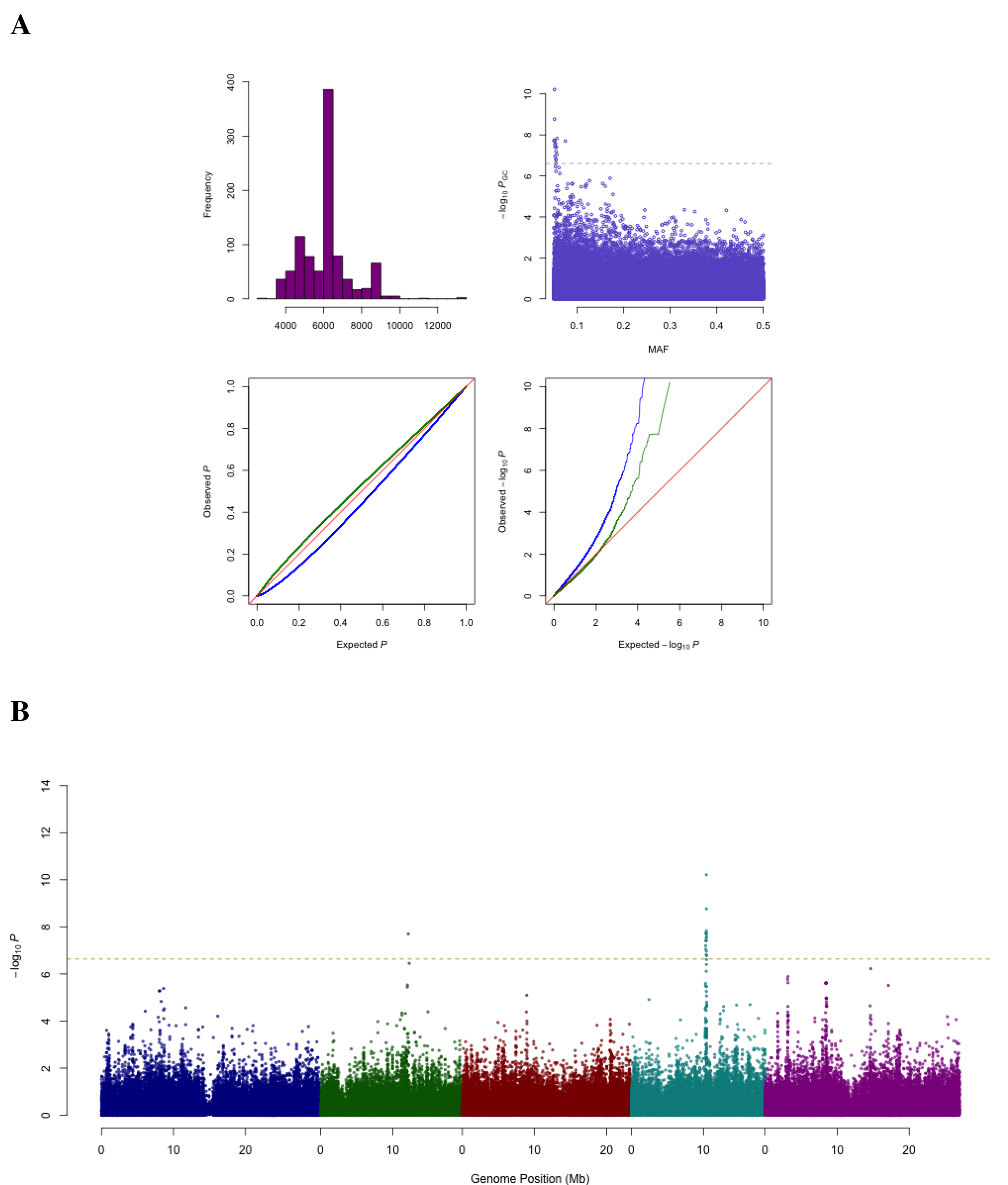

Supplement: S1 Figure — Summary of results for temperature seasonality. A: Phenotypic and p-value distributions. Top-left: phenotypic distribution; Top-right: -log10p-values after genomic control (GC) against minor allele frequencies (MAF); Bottom panels: Quantile-quantile plots of p-values and -log10p-values before (blue) and after (green) GC. B: Genome-wide association mapping for climate adaptability. The plotted -log10p-values are genomic controlled. Markers with minor allele frequencies less than 5% are removed. Chromosomes are distinguished by colors. The Bonferroni-corrected significance threshold is marked by the horizontal line. (TIF) [file pgen.1004842.s001.tif]

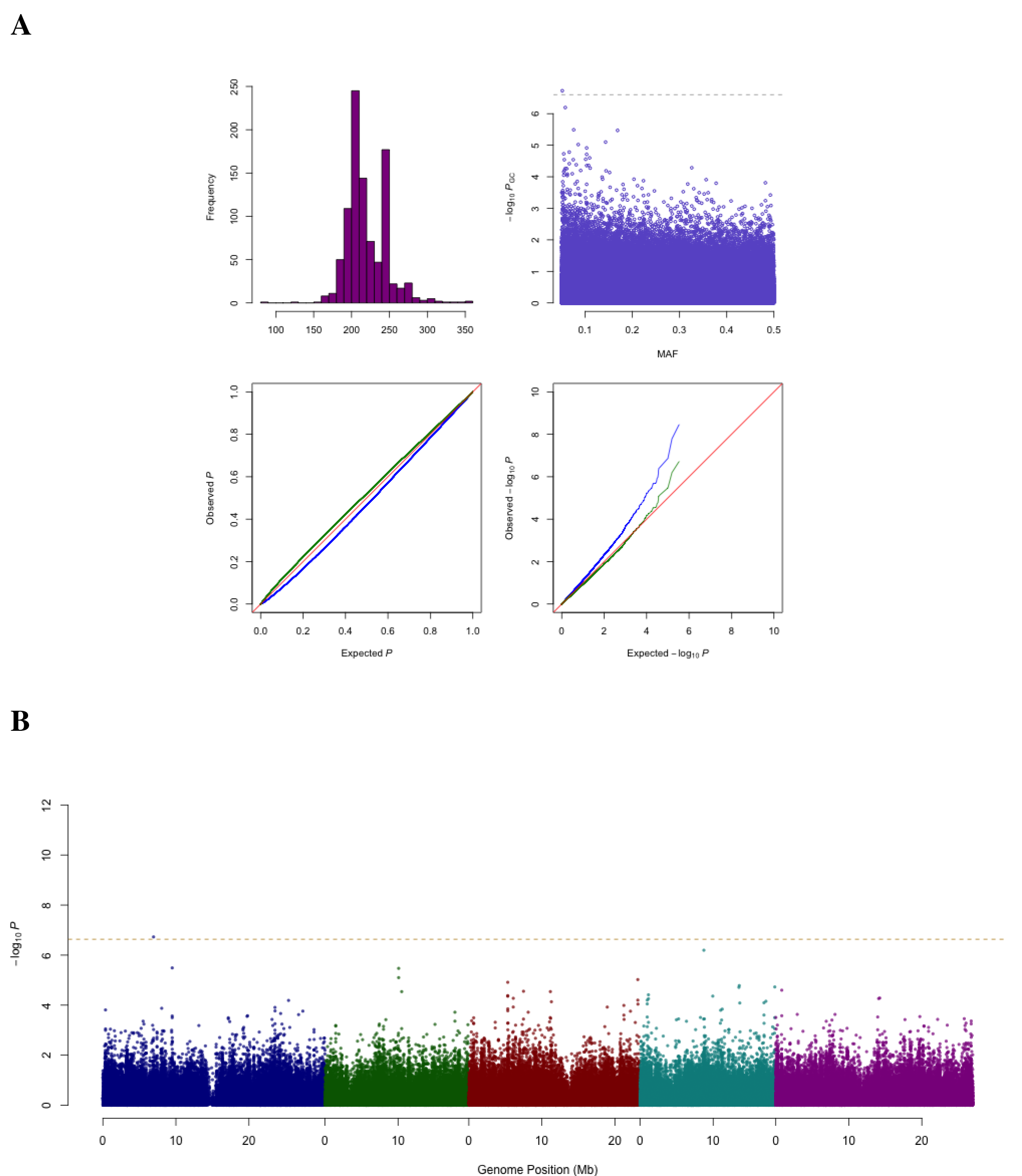

Supplement: S2 Figure — Summary of results for maximum temperature in the warmest month. A: Phenotypic and p-value distributions. Top-left: phenotypic distribution; Top-right: -log10p-values after genomic control (GC) against minor allele frequencies (MAF); Bottom panels: Quantile-quantile plots of p-values and -log10p-values before (blue) and after (green) GC. B: Genome-wide association mapping for climate adaptability. The plotted -log10p-values are genomic controlled. Markers with minor allele frequencies less than 5% are removed. Chromosomes are distinguished by colors. The Bonferroni-corrected significance threshold is marked by the horizontal line. (TIF) [file pgen.1004842.s002.tif]

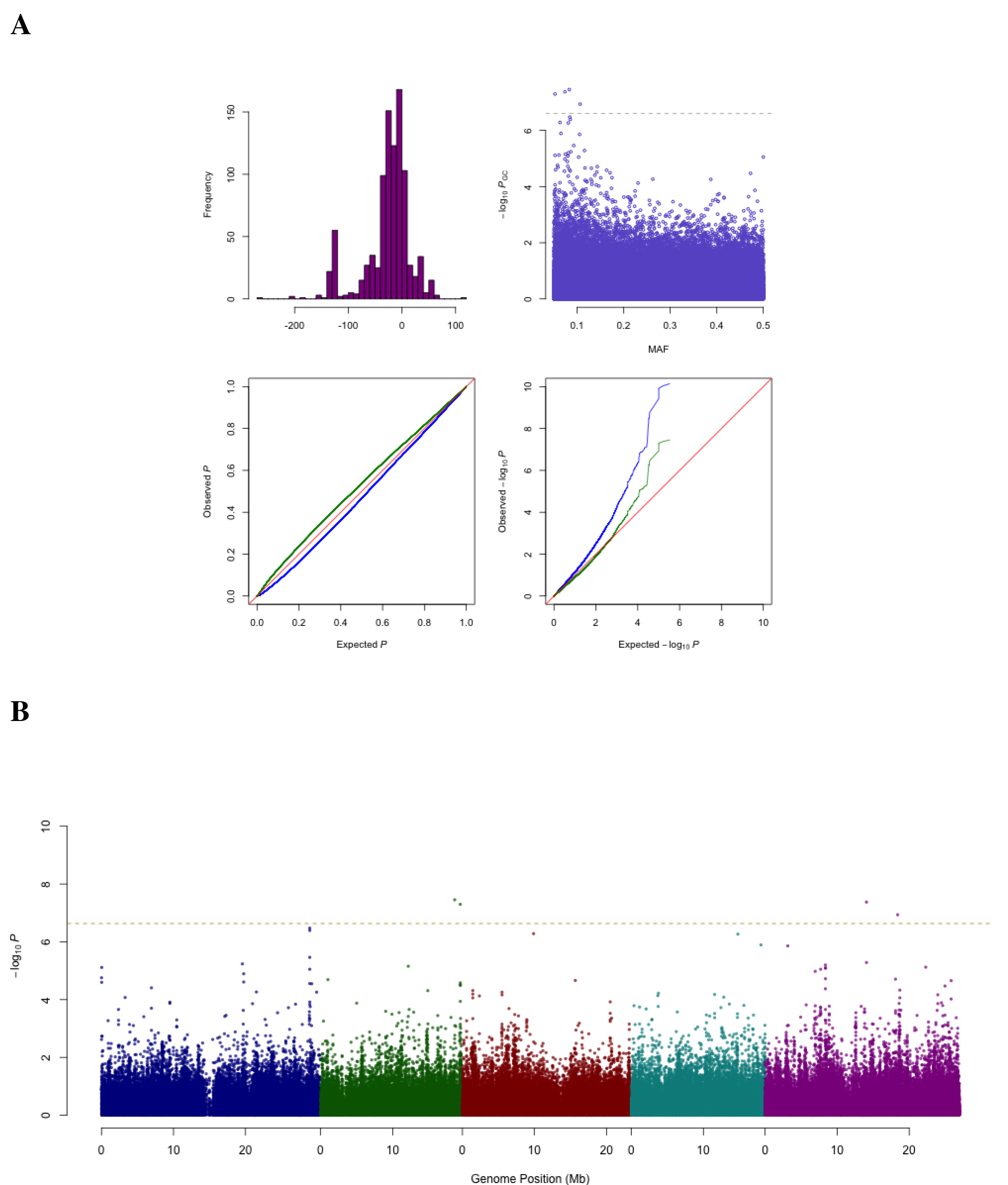

Supplement: S3 Figure — Summary of results for minimum temperature in the coldest month. A: Phenotypic and p-value distributions. Top-left: phenotypic distribution; Top-right: -log10p-values after genomic control (GC) against minor allele frequencies (MAF); Bottom panels: Quantile-quantile plots of p-values and -log10p-values before (blue) and after (green) GC. B: Genome-wide association mapping for climate adaptability. The plotted -log10p-values are genomic controlled. Markers with minor allele frequencies less than 5% are removed. Chromosomes are distinguished by colors. The Bonferroni-corrected significance threshold is marked by the horizontal line. (TIF) [file pgen.1004842.s003.tif]

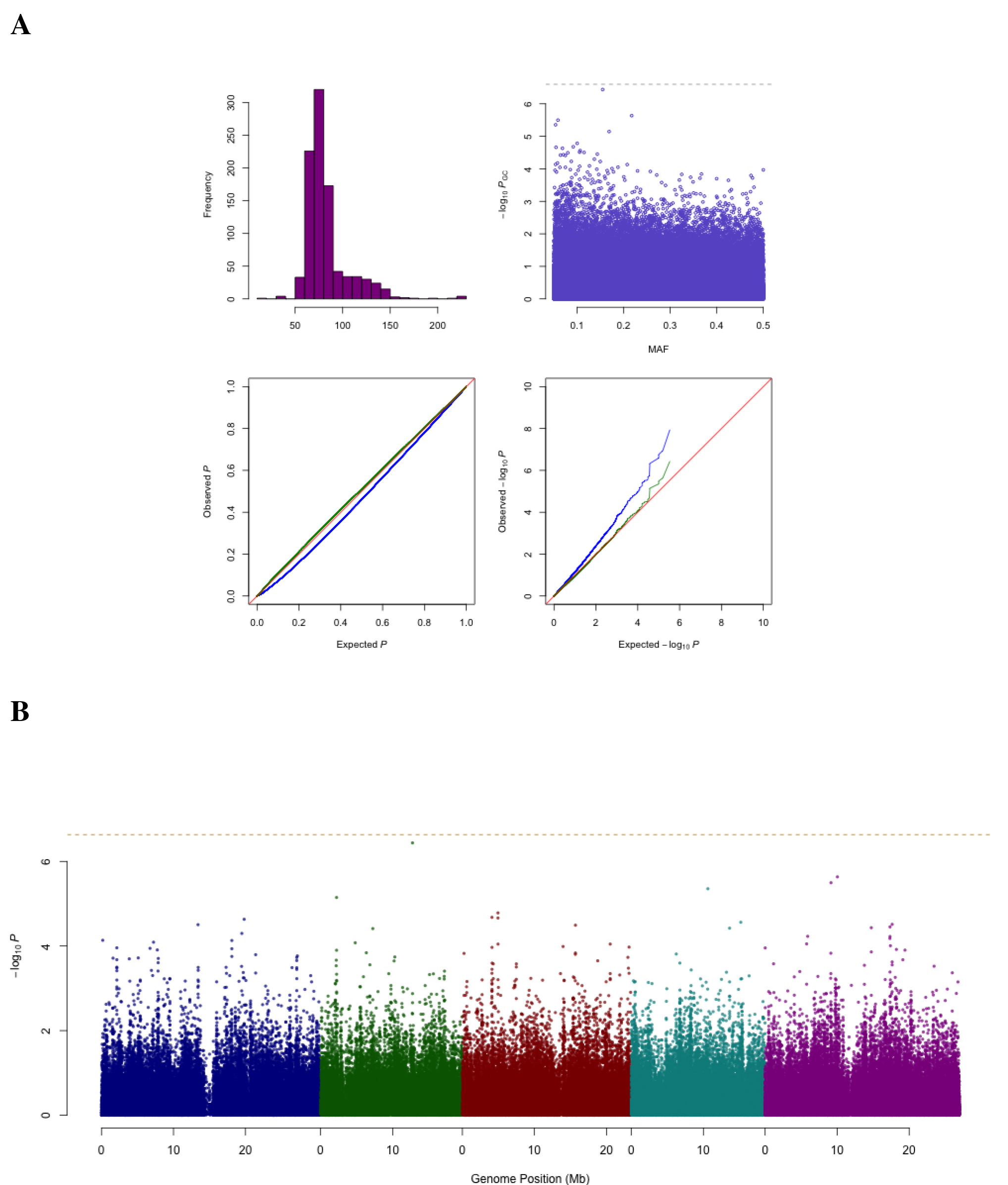

Supplement: S4 Figure — Summary of results for precipitation in the wettest month. A: Phenotypic and p-value distributions. Top-left: phenotypic distribution; Top-right: -log10p-values after genomic control (GC) against minor allele frequencies (MAF); Bottom panels: Quantile-quantile plots of p-values and -log10p-values before (blue) and after (green) GC. B: Genome-wide association mapping for climate adaptability. The plotted -log10p-values are genomic controlled. Markers with minor allele frequencies less than 5% are removed. Chromosomes are distinguished by colors. The Bonferroni-corrected significance threshold is marked by the horizontal line. (TIF) [file pgen.1004842.s004.tif]

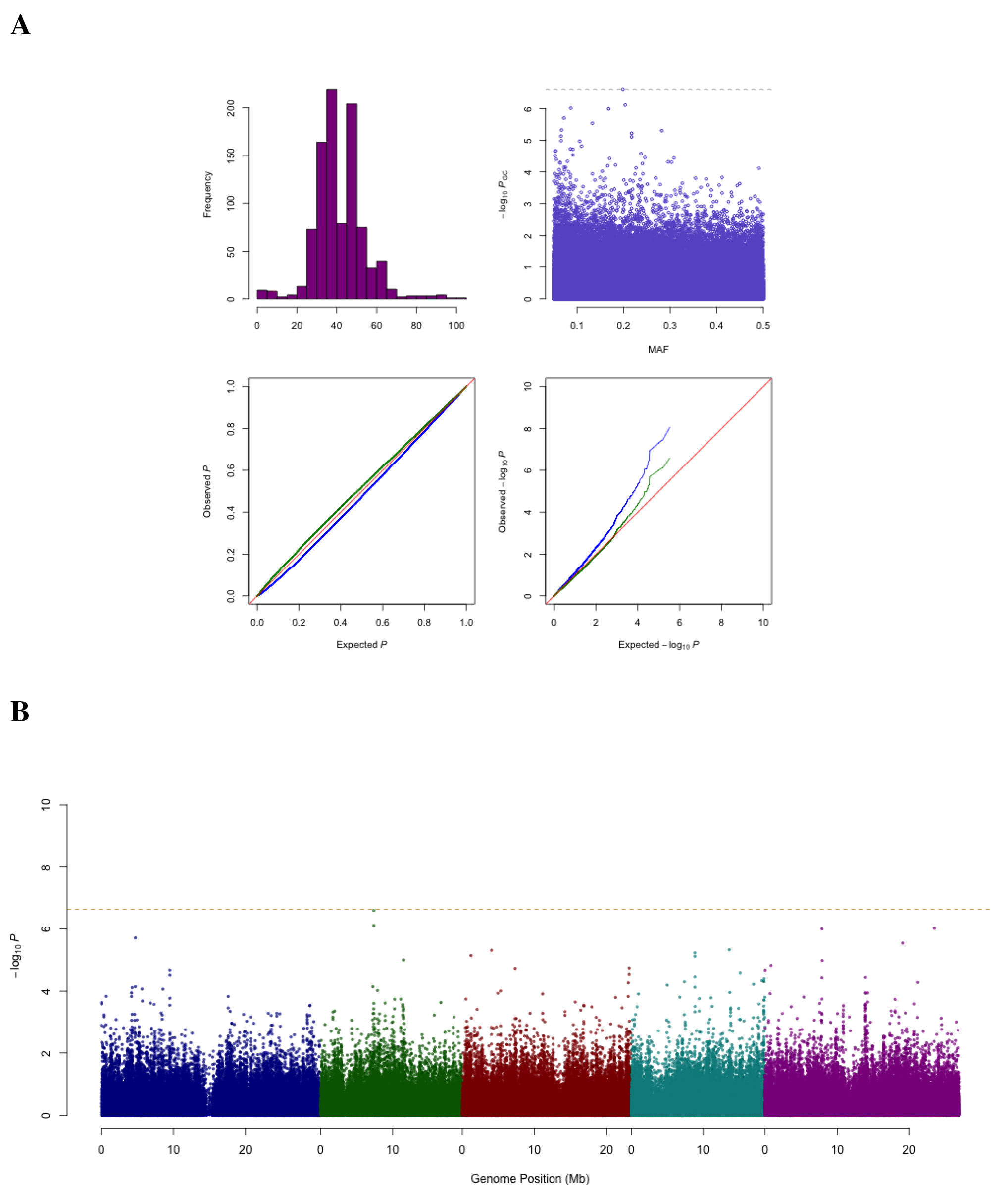

Supplement: S5 Figure — Summary of results for precipitation in the driest month. A: Phenotypic and p-value distributions. Top-left: phenotypic distribution; Top-right: -log10p-values after genomic control (GC) against minor allele frequencies (MAF); Bottom panels: Quantile-quantile plots of p-values and -log10p-values before (blue) and after (green) GC. B: Genome-wide association mapping for climate adaptability. The plotted -log10p-values are genomic controlled. Markers with minor allele frequencies less than 5% are removed. Chromosomes are distinguished by colors. The Bonferroni-corrected significance threshold is marked by the horizontal line. (TIF) [file pgen.1004842.s005.tif]

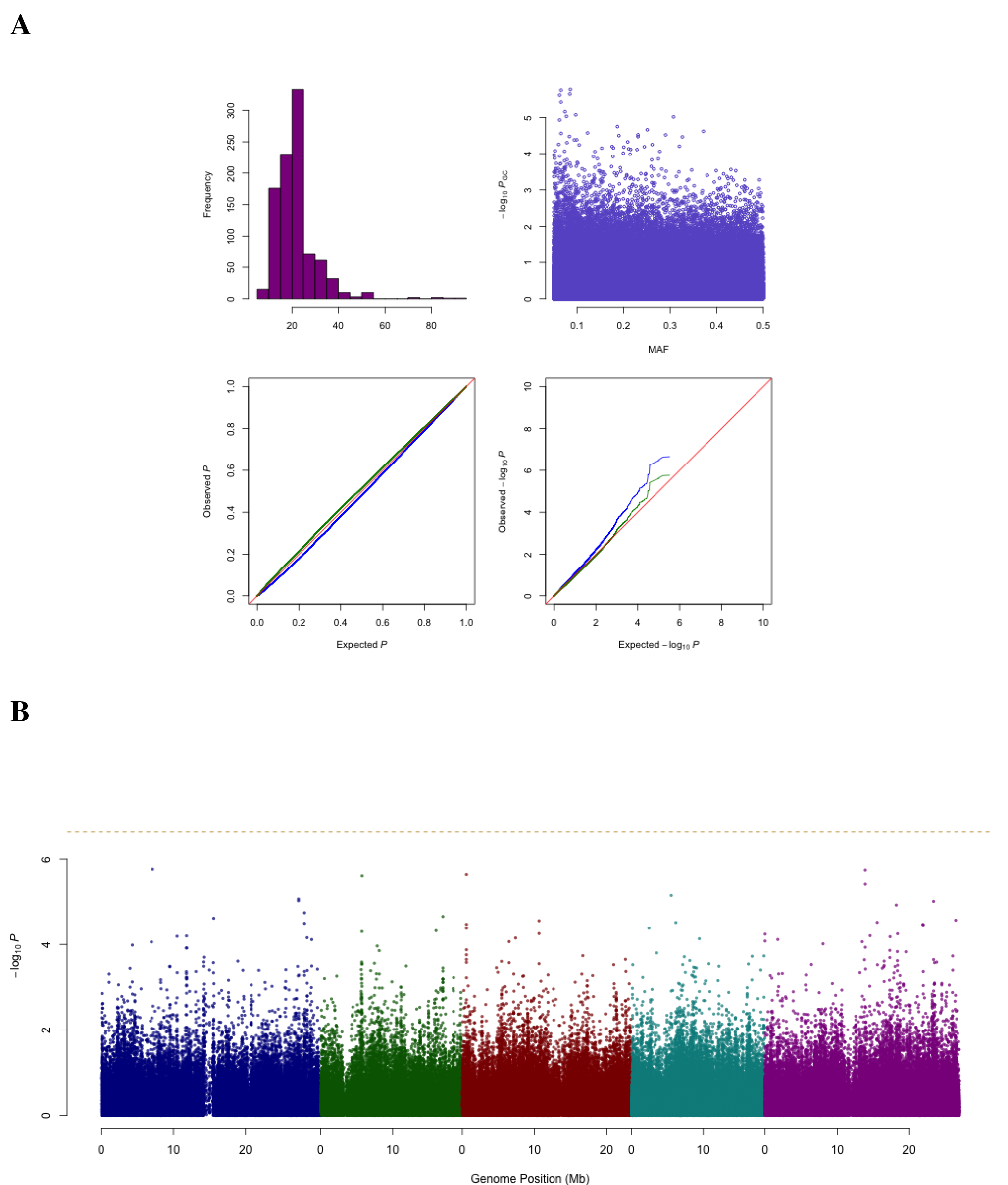

Supplement: S6 Figure — Summary of results for precipitation CV. A: Phenotypic and p-value distributions. Top-left: phenotypic distribution; Top-right: -log10p-values after genomic control (GC) against minor allele frequencies (MAF); Bottom panels: Quantile-quantile plots of p-values and -log10p-values before (blue) and after (green) GC. B: Genome-wide association mapping for climate adaptability. The plotted -log10p-values are genomic controlled. Markers with minor allele frequencies less than 5% are removed. Chromosomes are distinguished by colors. The Bonferroni-corrected significance threshold is marked by the horizontal line. (TIF) [file pgen.1004842.s006.tif]

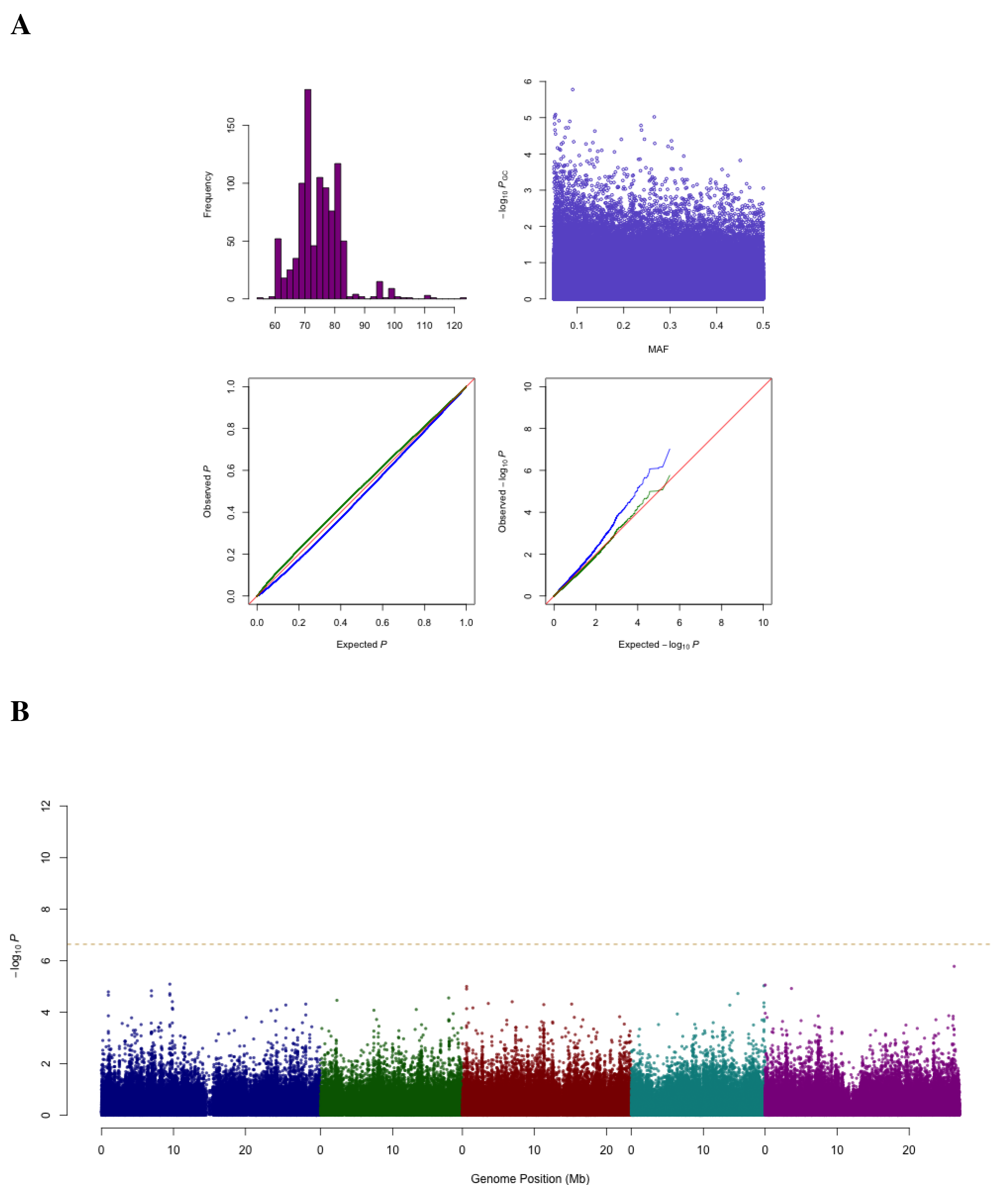

Supplement: S7 Figure — Summary of results for photosynthetically active radiation in spring. A: Phenotypic and p-value distributions. Top-left: phenotypic distribution; Top-right: -log10p-values after genomic control (GC) against minor allele frequencies (MAF); Bottom panels: Quantile-quantile plots of p-values and -log10p-values before (blue) and after (green) GC. B: Genome-wide association mapping for climate adaptability. The plotted -log10p-values are genomic controlled. Markers with minor allele frequencies less than 5% are removed. Chromosomes are distinguished by colors. The Bonferroni-corrected significance threshold is marked by the horizontal line. (TIF) [file pgen.1004842.s007.tif]

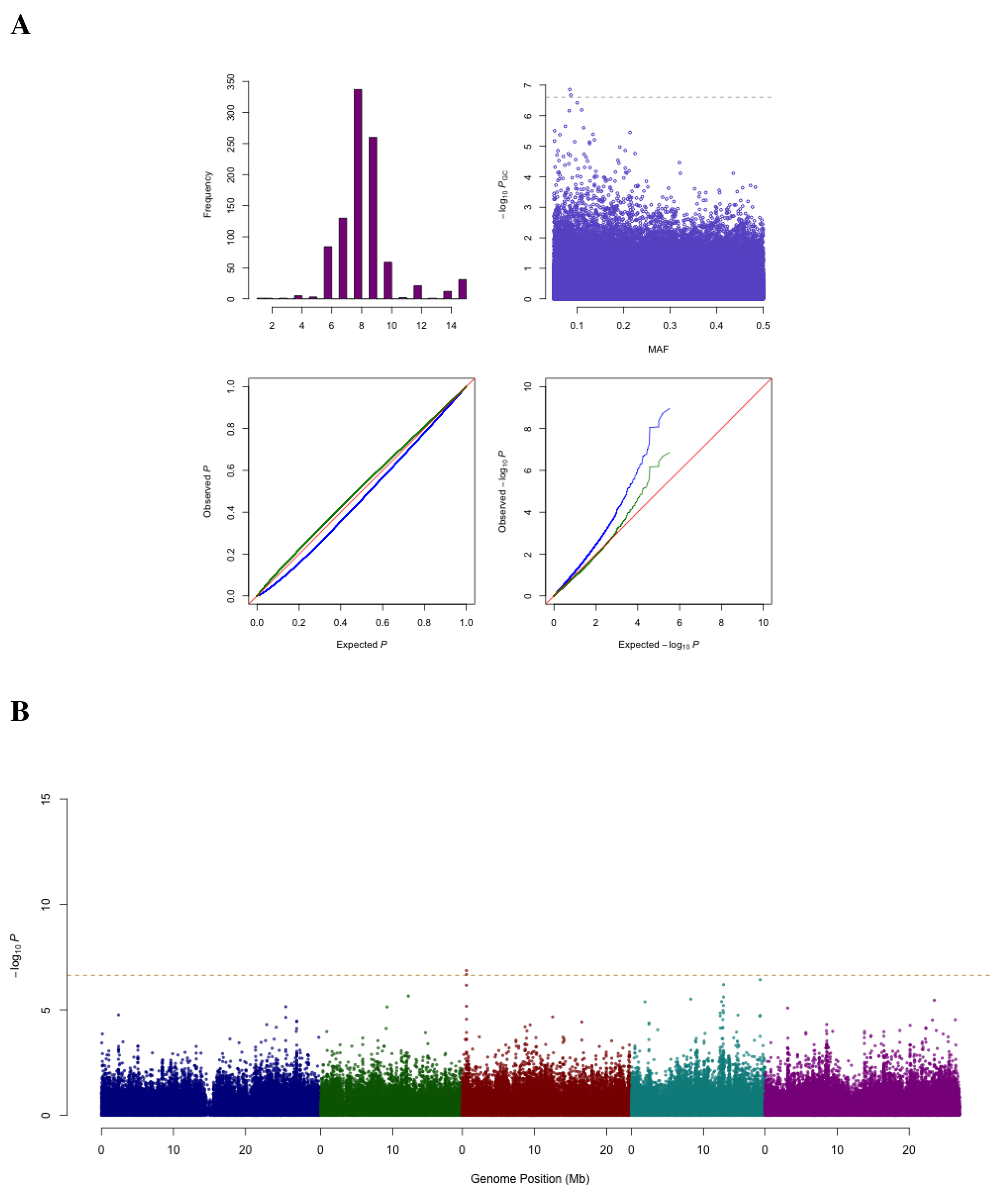

Supplement: S8 Figure — Summary of results for length of the growing season. A: Phenotypic and p-value distributions. Top-left: phenotypic distribution; Top-right: -log10p-values after genomic control (GC) against minor allele frequencies (MAF); Bottom panels: Quantile-quantile plots of p-values and -log10p-values before (blue) and after (green) GC. B: Genome-wide association mapping for climate adaptability. The plotted -log10p-values are genomic controlled. Markers with minor allele frequencies less than 5% are removed. Chromosomes are distinguished by colors. The Bonferroni-corrected significance threshold is marked by the horizontal line. (TIF) [file pgen.1004842.s008.tif]

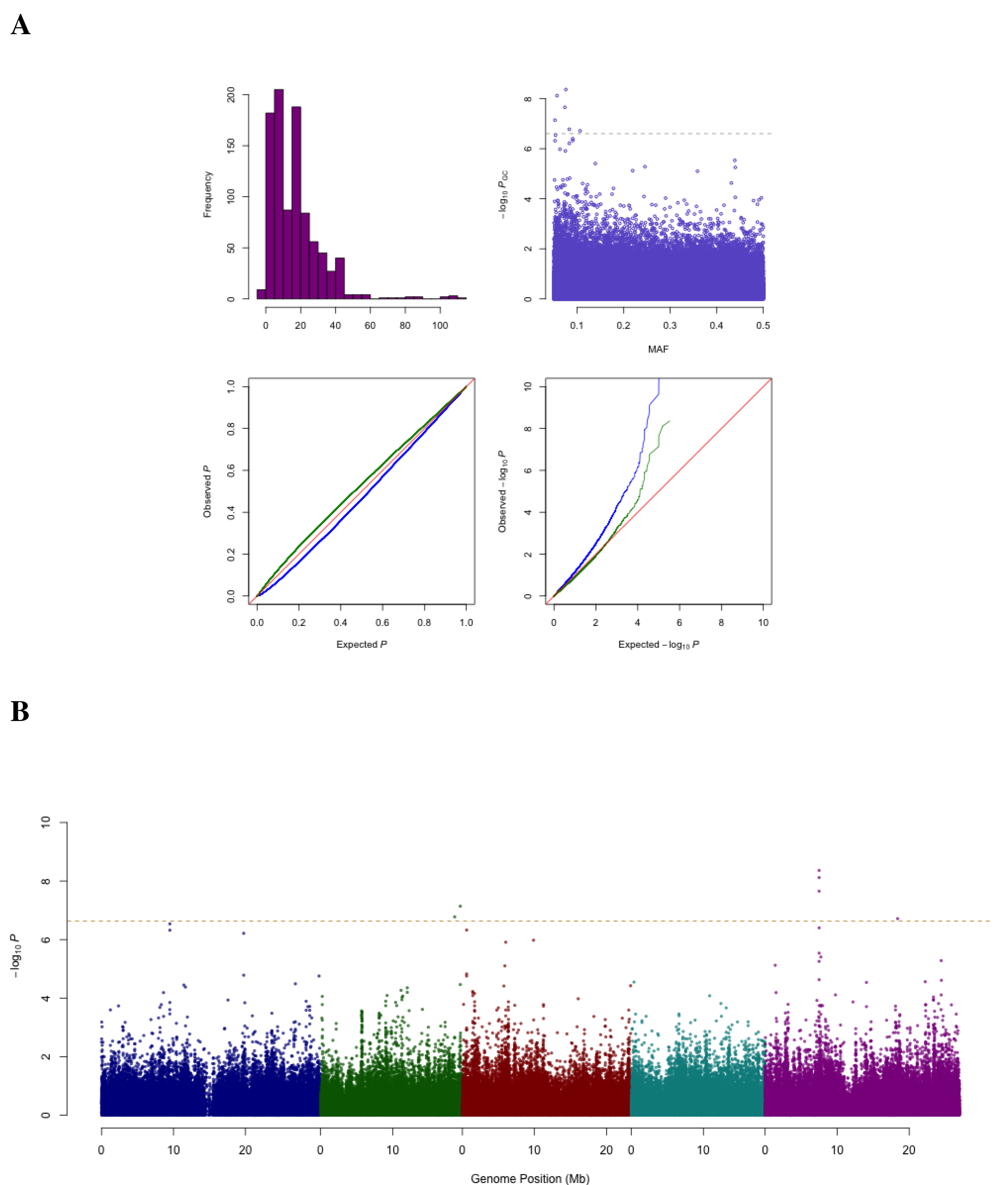

Supplement: S9 Figure — Summary of results for number of consecutive cold days. A: Phenotypic and p-value distributions. Top-left: phenotypic distribution; Top-right: -log10p-values after genomic control (GC) against minor allele frequencies (MAF); Bottom panels: Quantile-quantile plots of p-values and -log10p-values before (blue) and after (green) GC. B: Genome-wide association mapping for climate adaptability. The plotted -log10p-values are genomic controlled. Markers with minor allele frequencies less than 5% are removed. Chromosomes are distinguished by colors. The Bonferroni-corrected significance threshold is marked by the horizontal line. (TIF) [file pgen.1004842.s009.tif]

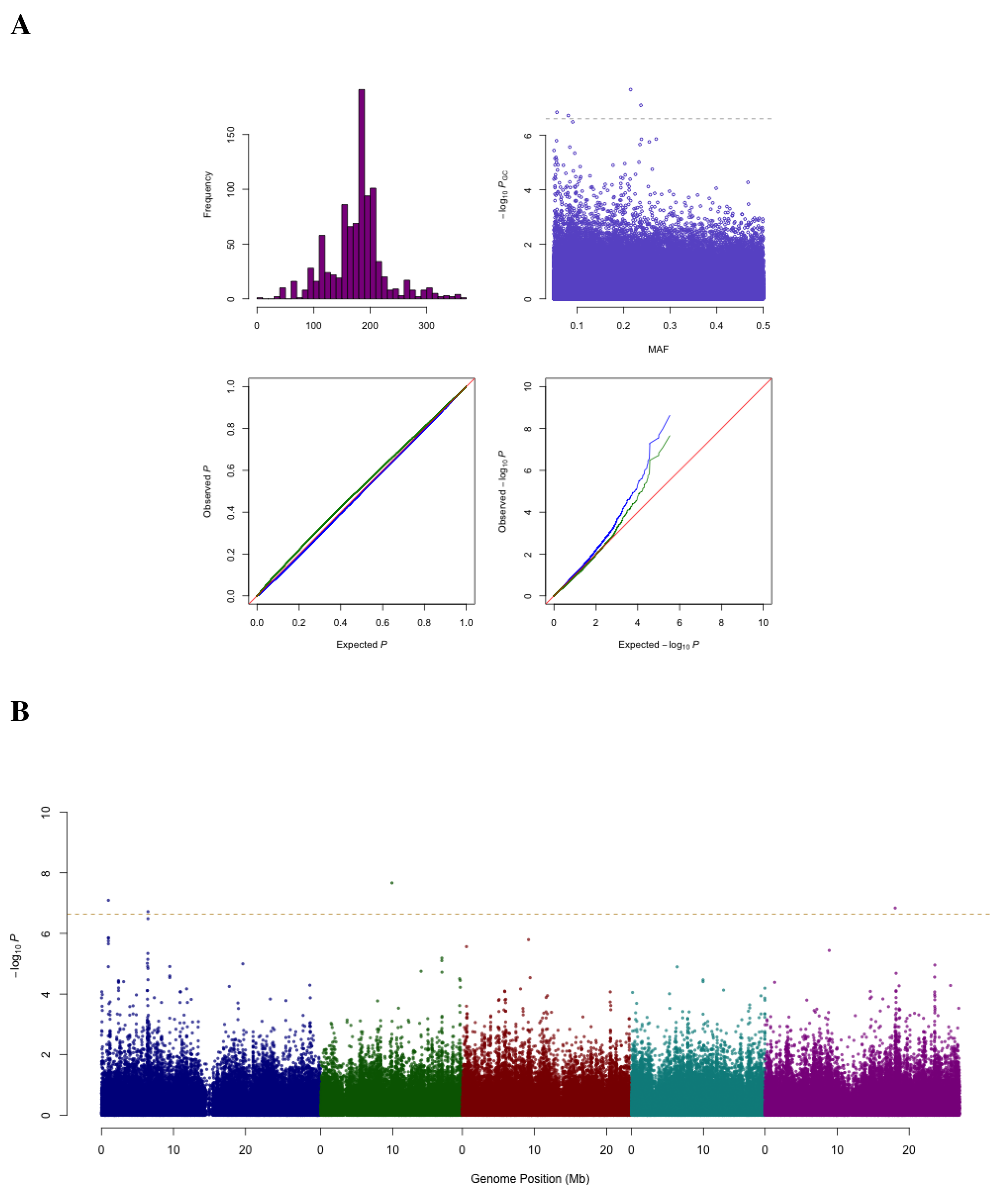

Supplement: S10 Figure — Summary of results for number of consecutive frost-free days. A: Phenotypic and p-value distributions. Top-left: phenotypic distribution; Top-right: -log10p-values after genomic control (GC) against minor allele frequencies (MAF); Bottom panels: Quantile-quantile plots of p-values and -log10p-values before (blue) and after (green) GC. B: Genome-wide association mapping for climate adaptability. The plotted -log10p-values are genomic controlled. Markers with minor allele frequencies less than 5% are removed. Chromosomes are distinguished by colors. The Bonferroni-corrected significance threshold is marked by the horizontal line. (TIF) [file pgen.1004842.s010.tif]

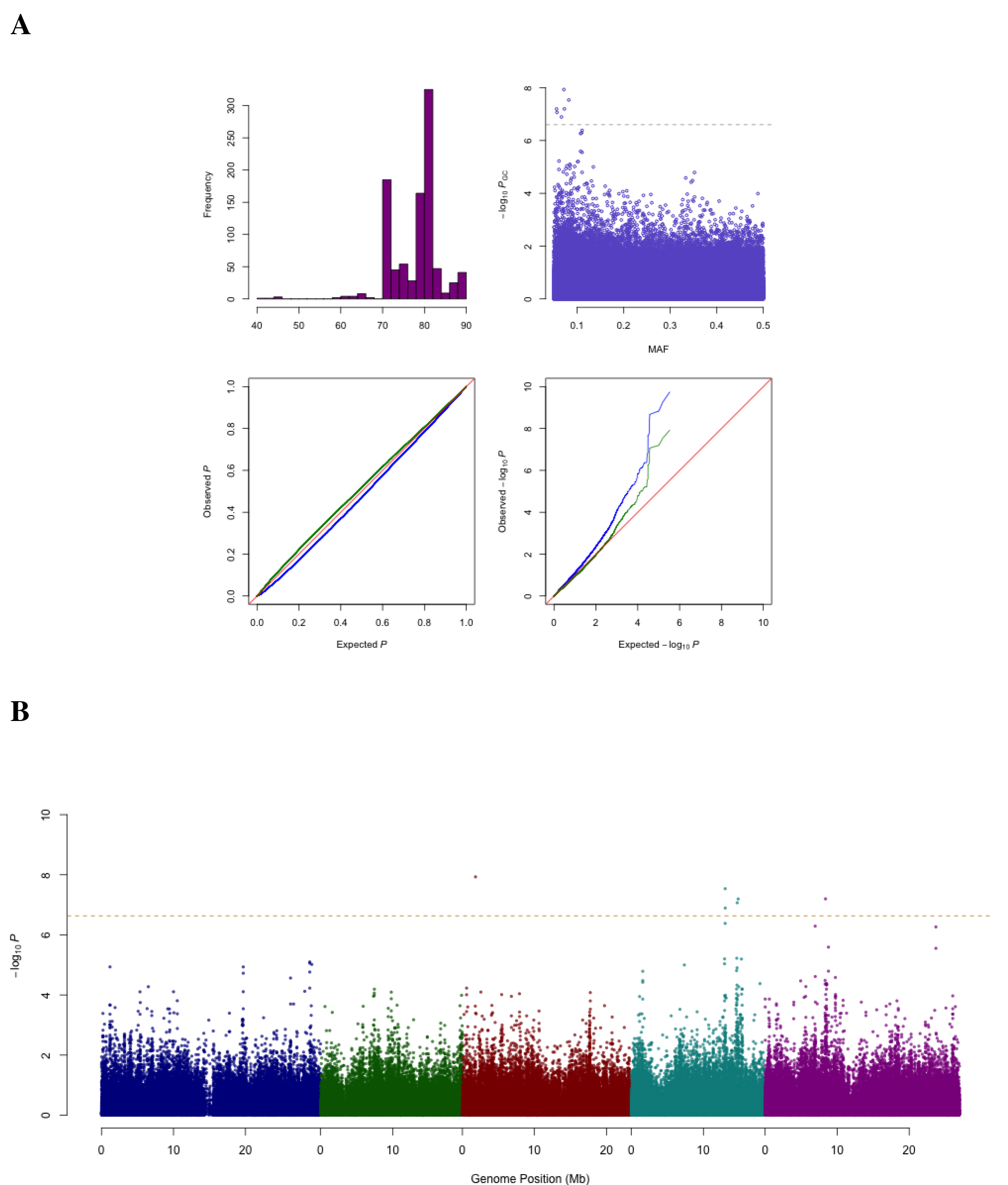

Supplement: S11 Figure — Summary of results for relative humidity in spring. A: Phenotypic and p-value distributions. Top-left: phenotypic distribution; Top-right: -log10p-values after genomic control (GC) against minor allele frequencies (MAF); Bottom panels: Quantile-quantile plots of p-values and -log10p-values before (blue) and after (green) GC. B: Genome-wide association mapping for climate adaptability. The plotted -log10p-values are genomic controlled. Markers with minor allele frequencies less than 5% are removed. Chromosomes are distinguished by colors. The Bonferroni-corrected significance threshold is marked by the horizontal line. (TIF) [file pgen.1004842.s011.tif]

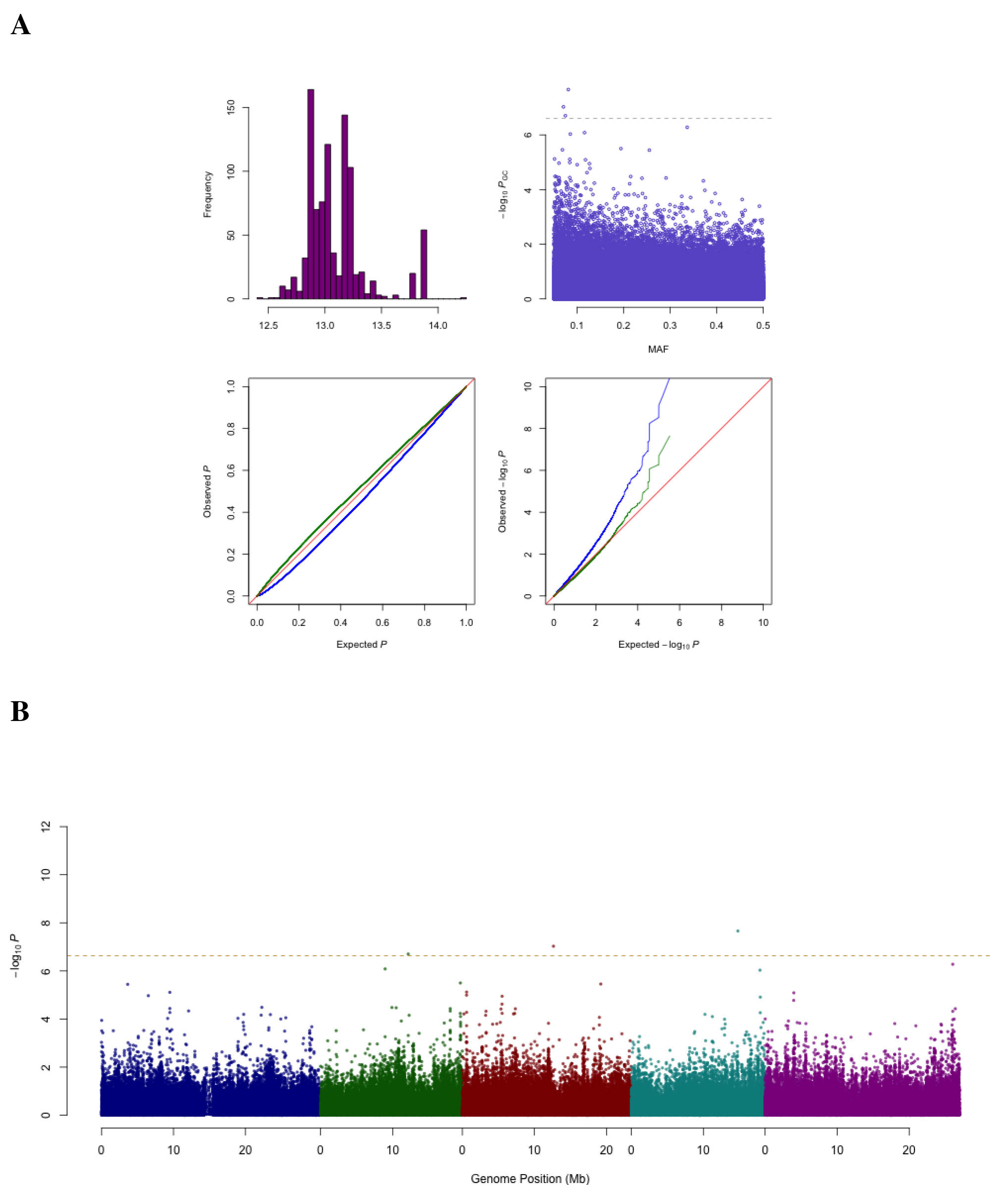

Supplement: S12 Figure — Summary of results for day-length in spring. A: Phenotypic and p-value distributions. Top-left: phenotypic distribution; Top-right: -log10p-values after genomic control (GC) against minor allele frequencies (MAF); Bottom panels: Quantile-quantile plots of p-values and -log10p-values before (blue) and after (green) GC. B: Genome-wide association mapping for climate adaptability. The plotted -log10p-values are genomic controlled. Markers with minor allele frequencies less than 5% are removed. Chromosomes are distinguished by colors. The Bonferroni-corrected significance threshold is marked by the horizontal line. (TIF) [file pgen.1004842.s012.tif]

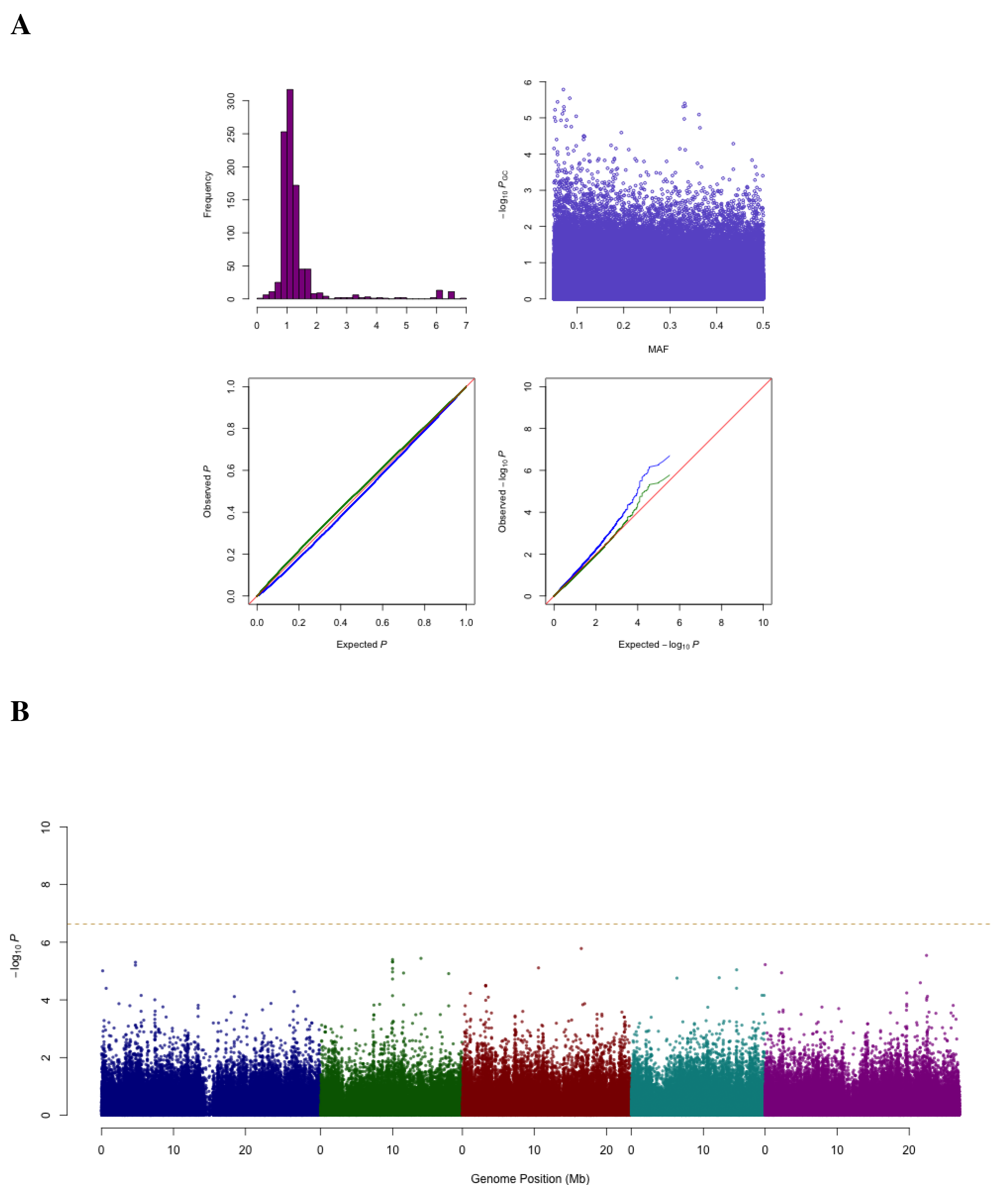

Supplement: S13 Figure — Summary of results for aridity index. A: Phenotypic and p-value distributions. Top-left: phenotypic distribution; Top-right: -log10p-values after genomic control (GC) against minor allele frequencies (MAF); Bottom panels: Quantile-quantile plots of p-values and -log10p-values before (blue) and after (green) GC. B: Genome-wide association mapping for climate adaptability. The plotted -log10p-values are genomic controlled. Markers with minor allele frequencies less than 5% are removed. Chromosomes are distinguished by colors. The Bonferroni-corrected significance threshold is marked by the horizontal line. (TIF) [file pgen.1004842.s013.tif]

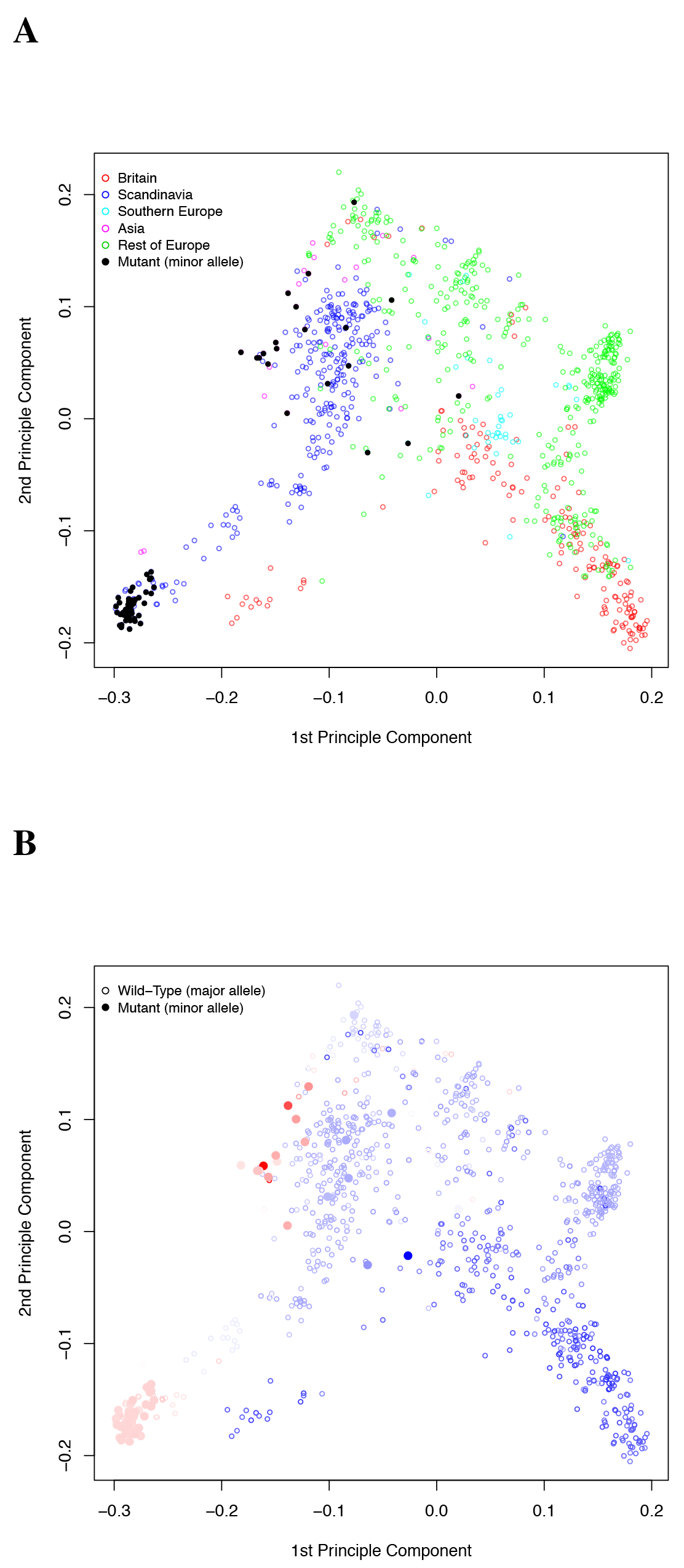

Supplement: S14 Figure — Principle components of the genomic kinship for the two alleles on chromosome 2 at 12,169,701 bp. Corresponding climate variable: temperature seasonality. A: Genomic kinship principle components categorized based on geographical regions. B: Genomic kinship principle components colored based on the scale of the climate variable. The colors scale from pure blue (the minimum climate variable value) to pure red (the maximum value). (TIF) [file pgen.1004842.s014.tif]

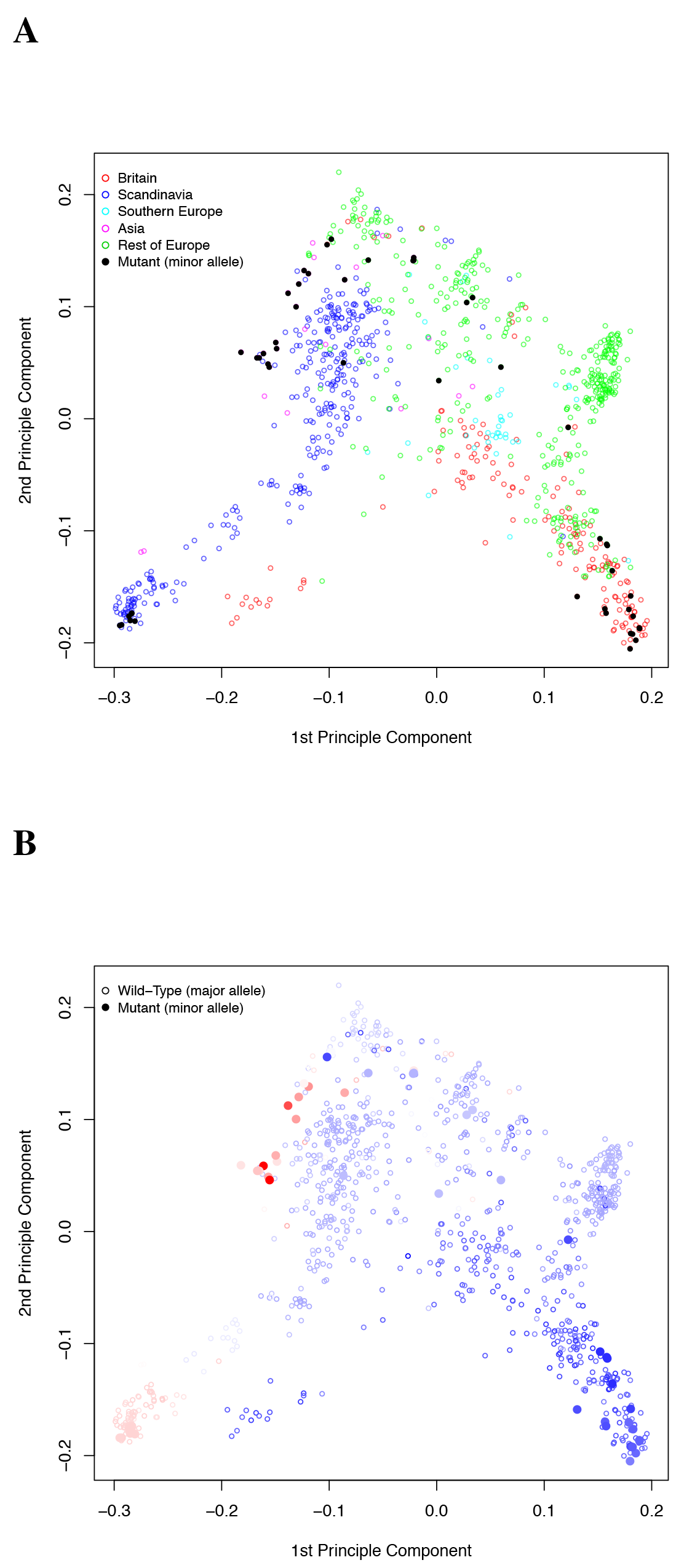

Supplement: S15 Figure — Principle components of the genomic kinship for the two alleles on chromosome 4 at 10,406,018 bp. Corresponding climate variable: temperature seasonality. A: Genomic kinship principle components categorized based on geographical regions. B: Genomic kinship principle components colored based on the scale of the climate variable. The colors scale from pure blue (the minimum climate variable value) to pure red (the maximum value). (TIF) [file pgen.1004842.s015.tif]

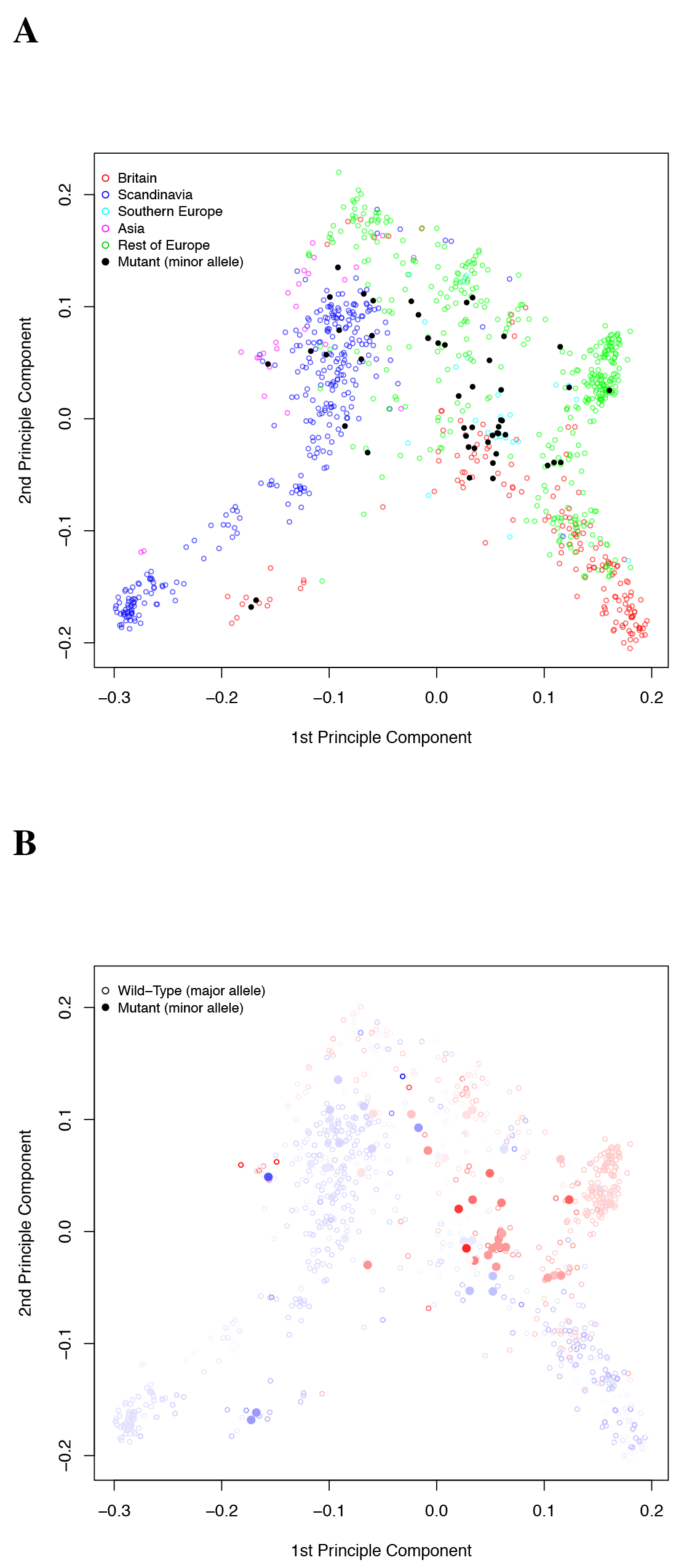

Supplement: S16 Figure — Principle components of the genomic kinship for the two alleles on chromosome 1 at 6,936,457 bp. Corresponding climate variable: maximum temperature in the warmest month. A: Genomic kinship principle components categorized based on geographical regions. B: Genomic kinship principle components colored based on the scale of the climate variable. The colors scale from pure blue (the minimum climate variable value) to pure red (the maximum value). (TIF) [file pgen.1004842.s016.tif]

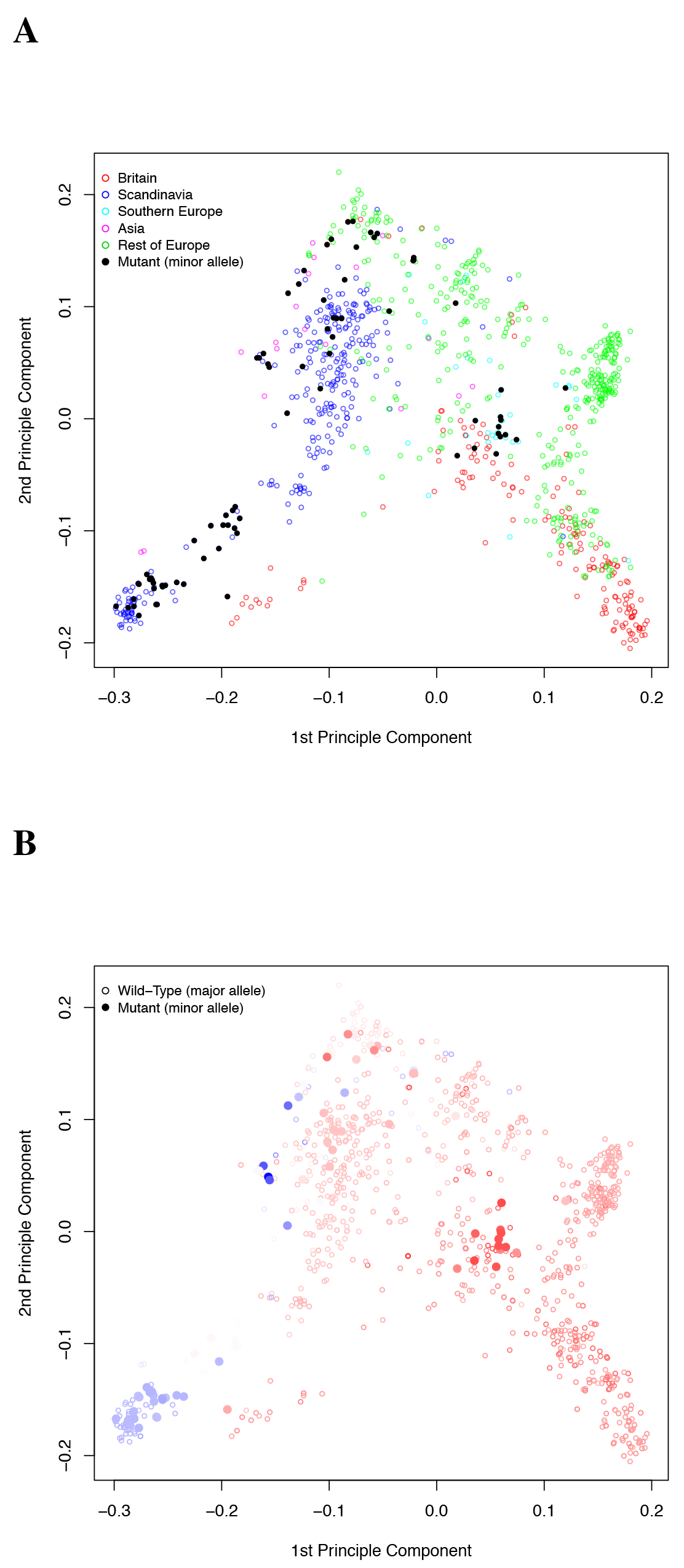

Supplement: S17 Figure — Principle components of the genomic kinship for the two alleles on chromosome 2 at 18,620,697 bp. Corresponding climate variable: minimum temperature in the coldest month. A: Genomic kinship principle components categorized based on geographical regions. B: Genomic kinship principle components colored based on the scale of the climate variable. The colors scale from pure blue (the minimum climate variable value) to pure red (the maximum value). (TIF) [file pgen.1004842.s017.tif]

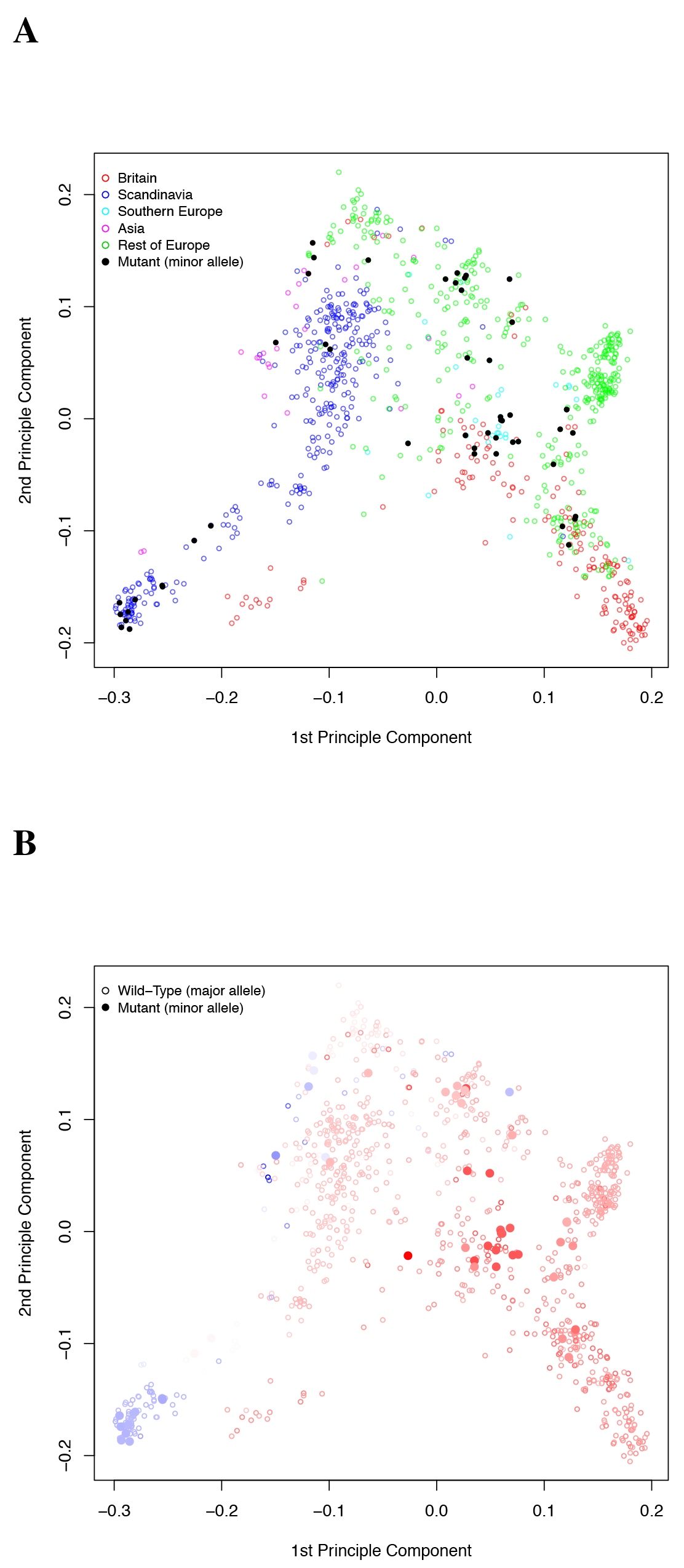

Supplement: S18 Figure — Principle components of the genomic kinship for the two alleles on chromosome 2 at 19,397,389 bp. Corresponding climate variable: minimum temperature in the coldest month. A: Genomic kinship principle components categorized based on geographical regions. B: Genomic kinship principle components colored based on the scale of the climate variable. The colors scale from pure blue (the minimum climate variable value) to pure red (the maximum value). (TIF) [file pgen.1004842.s018.tif]

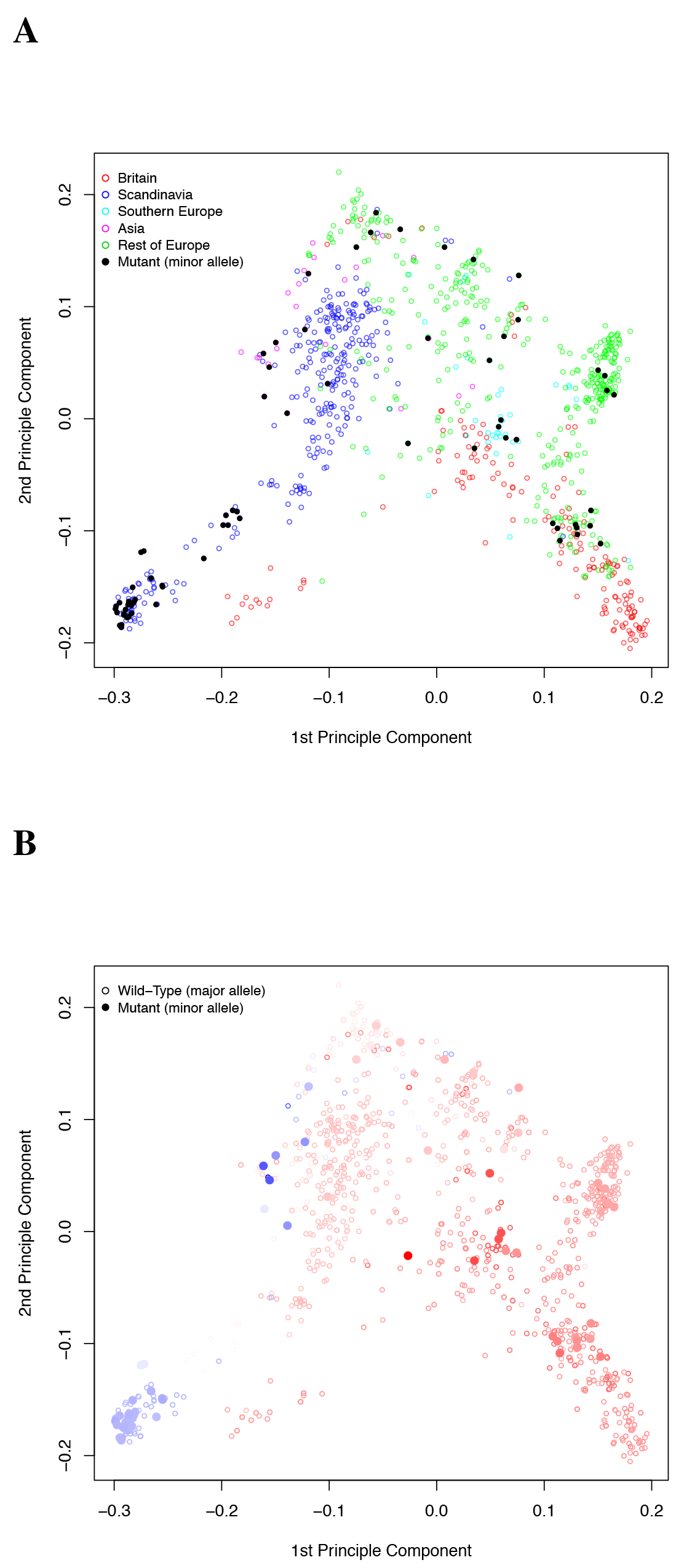

Supplement: S19 Figure — Principle components of the genomic kinship for the two alleles on chromosome 5 at 14,067,526 bp. Corresponding climate variable: minimum temperature in the coldest month. A: Genomic kinship principle components categorized based on geographical regions. B: Genomic kinship principle components colored based on the scale of the climate variable. The colors scale from pure blue (the minimum climate variable value) to pure red (the maximum value). (TIF) [file pgen.1004842.s019.tif]

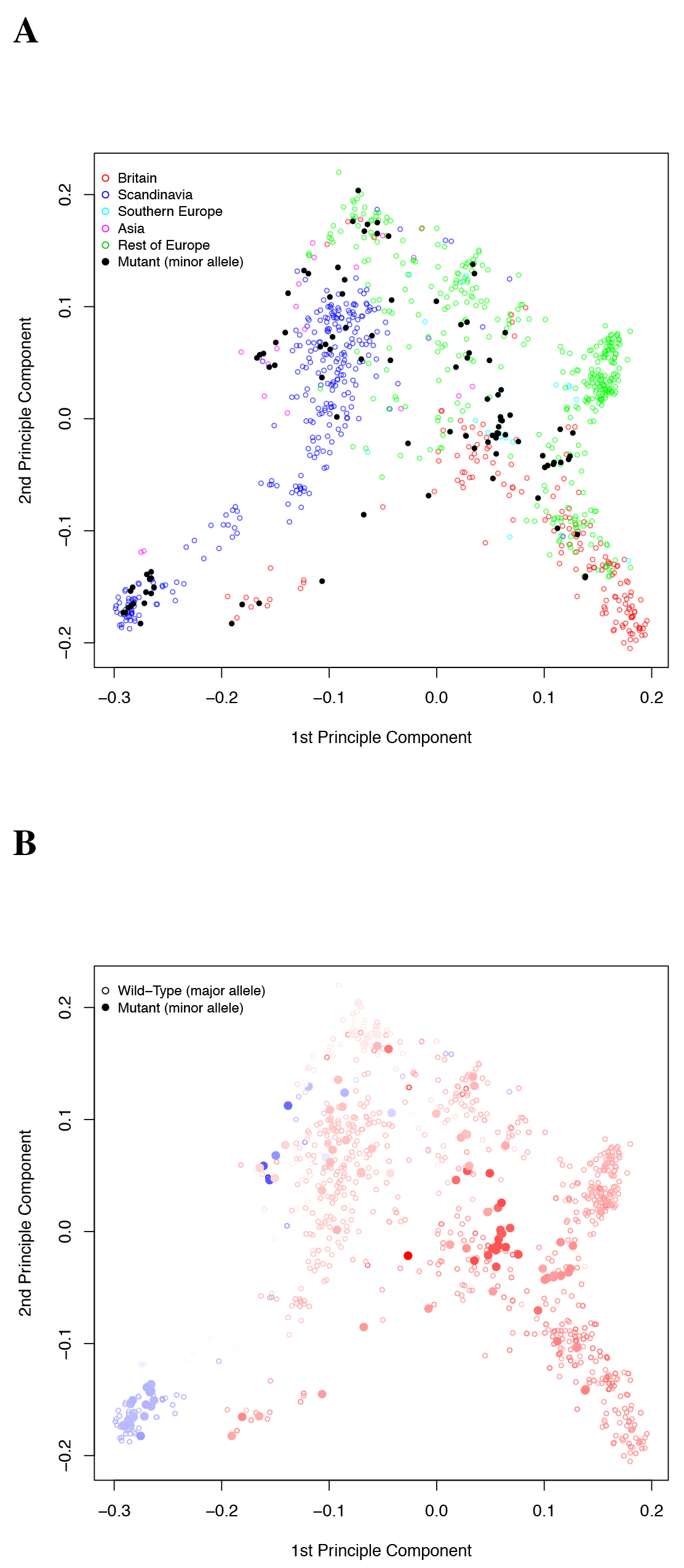

Supplement: S20 Figure — Principle components of the genomic kinship for the two alleles on chromosome 5 at 18,397,418 bp. Corresponding climate variable: minimum temperature in the coldest month. A: Genomic kinship principle components categorized based on geographical regions. B: Genomic kinship principle components colored based on the scale of the climate variable. The colors scale from pure blue (the minimum climate variable value) to pure red (the maximum value). (TIF) [file pgen.1004842.s020.tif]

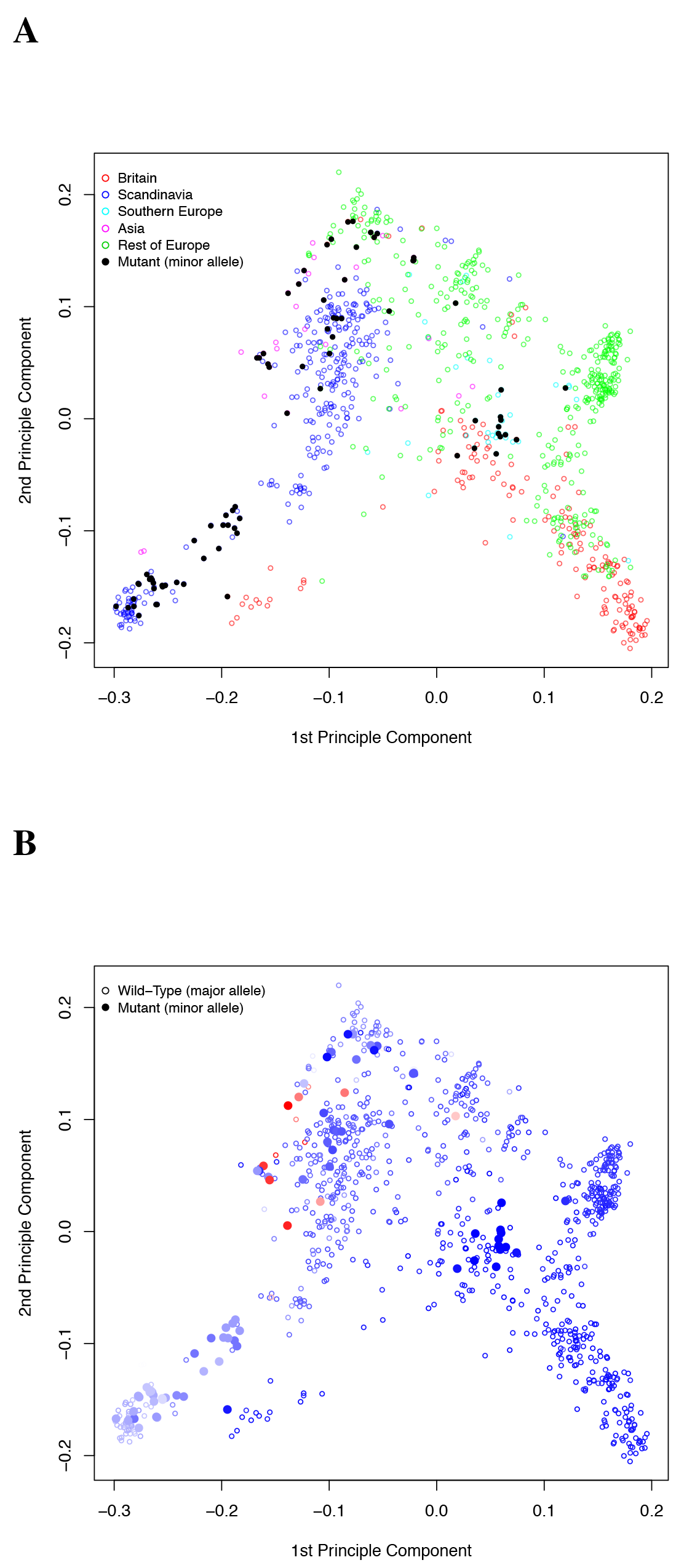

Supplement: S21 Figure — Principle components of the genomic kinship for the two alleles on chromosome 2 at 18,620,697 bp. Corresponding climate variable: number of consecutive cold days. A: Genomic kinship principle components categorized based on geographical regions. B: Genomic kinship principle components colored based on the scale of the climate variable. The colors scale from pure blue (the minimum climate variable value) to pure red (the maximum value). (TIF) [file pgen.1004842.s021.tif]

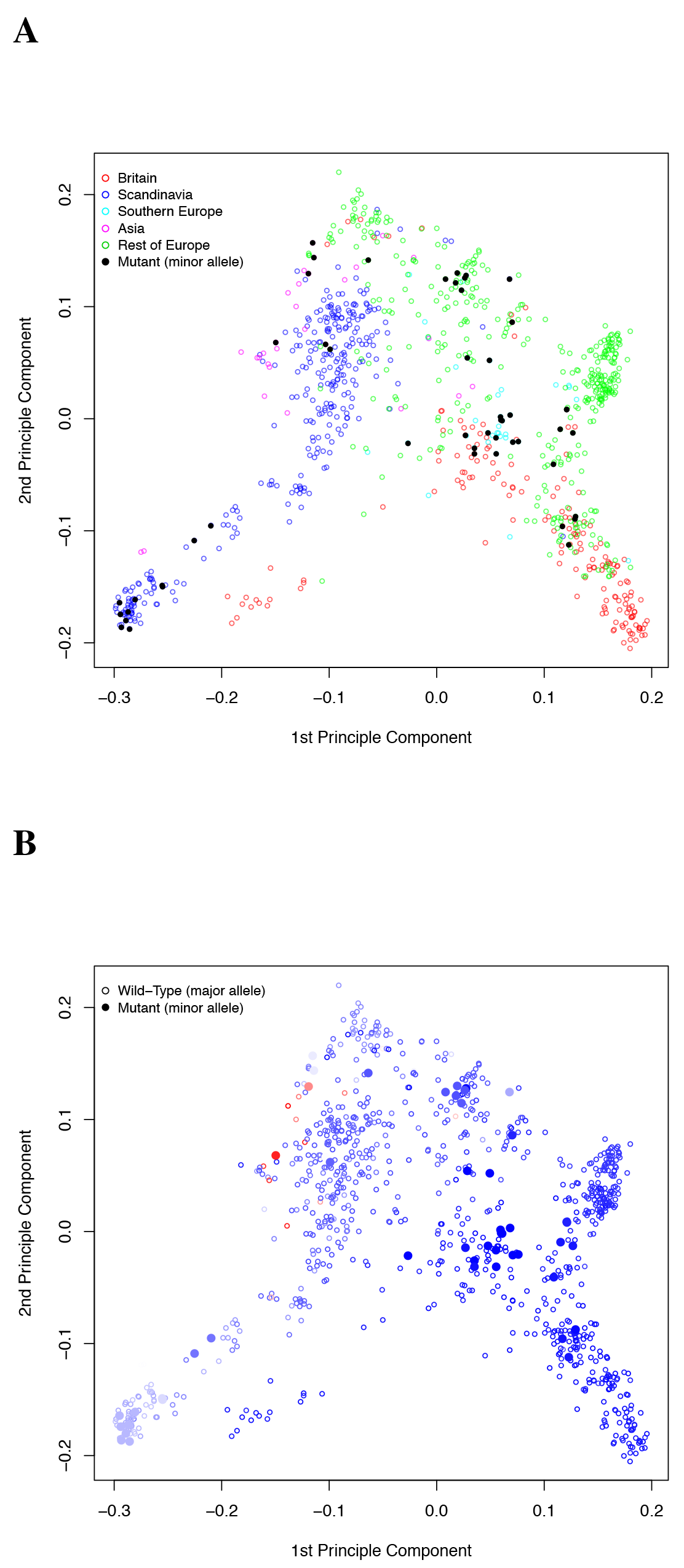

Supplement: S22 Figure — Principle components of the genomic kinship for the two alleles on chromosome 2 at 19,397,389 bp. Corresponding climate variable: number of consecutive cold days. A: Genomic kinship principle components categorized based on geographical regions. B: Genomic kinship principle components colored based on the scale of the climate variable. The colors scale from pure blue (the minimum climate variable value) to pure red (the maximum value). (TIF) [file pgen.1004842.s022.tif]

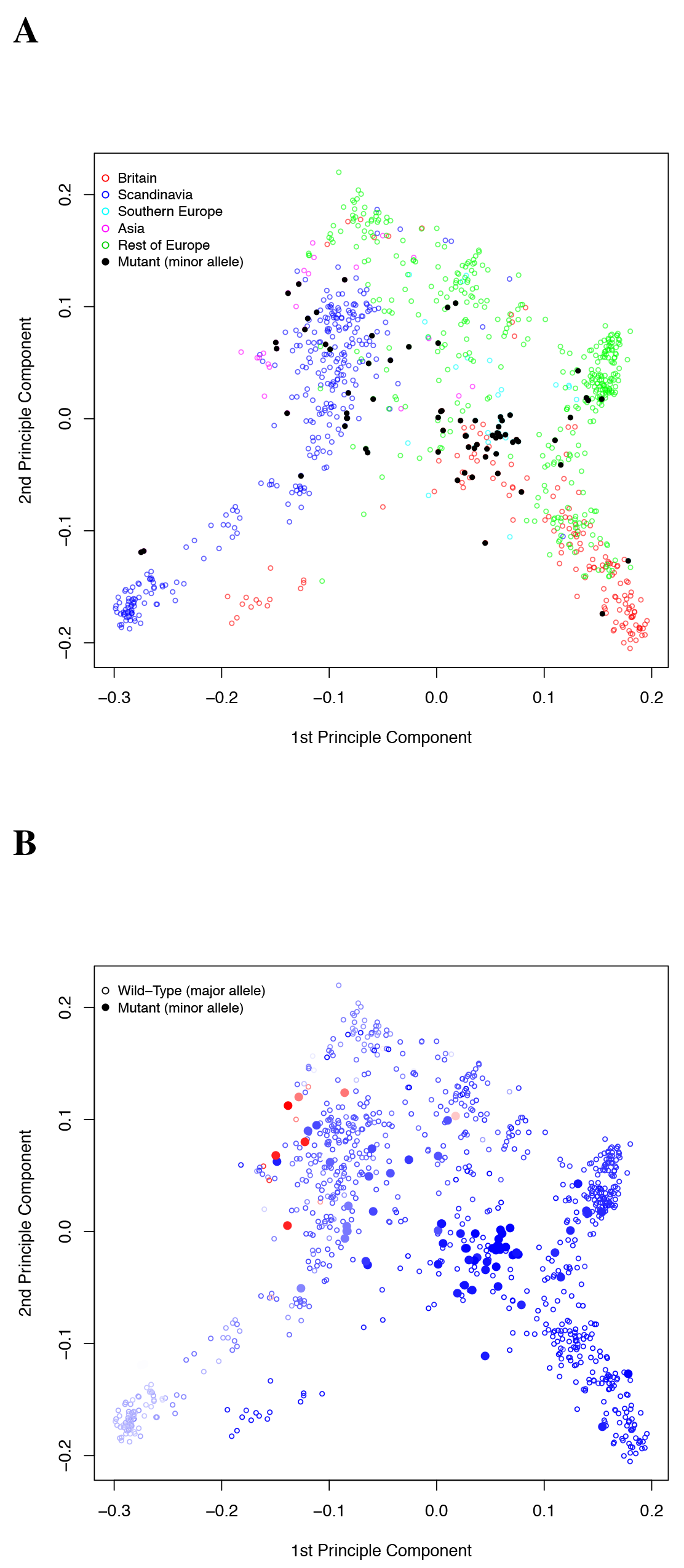

Supplement: S23 Figure — Principle components of the genomic kinship for the two alleles on chromosome 5 at 7,492,277 bp. Corresponding climate variable: number of consecutive cold days. A: Genomic kinship principle components categorized based on geographical regions. B: Genomic kinship principle components colored based on the scale of the climate variable. The colors scale from pure blue (the minimum climate variable value) to pure red (the maximum value). (TIF) [file pgen.1004842.s023.tif]

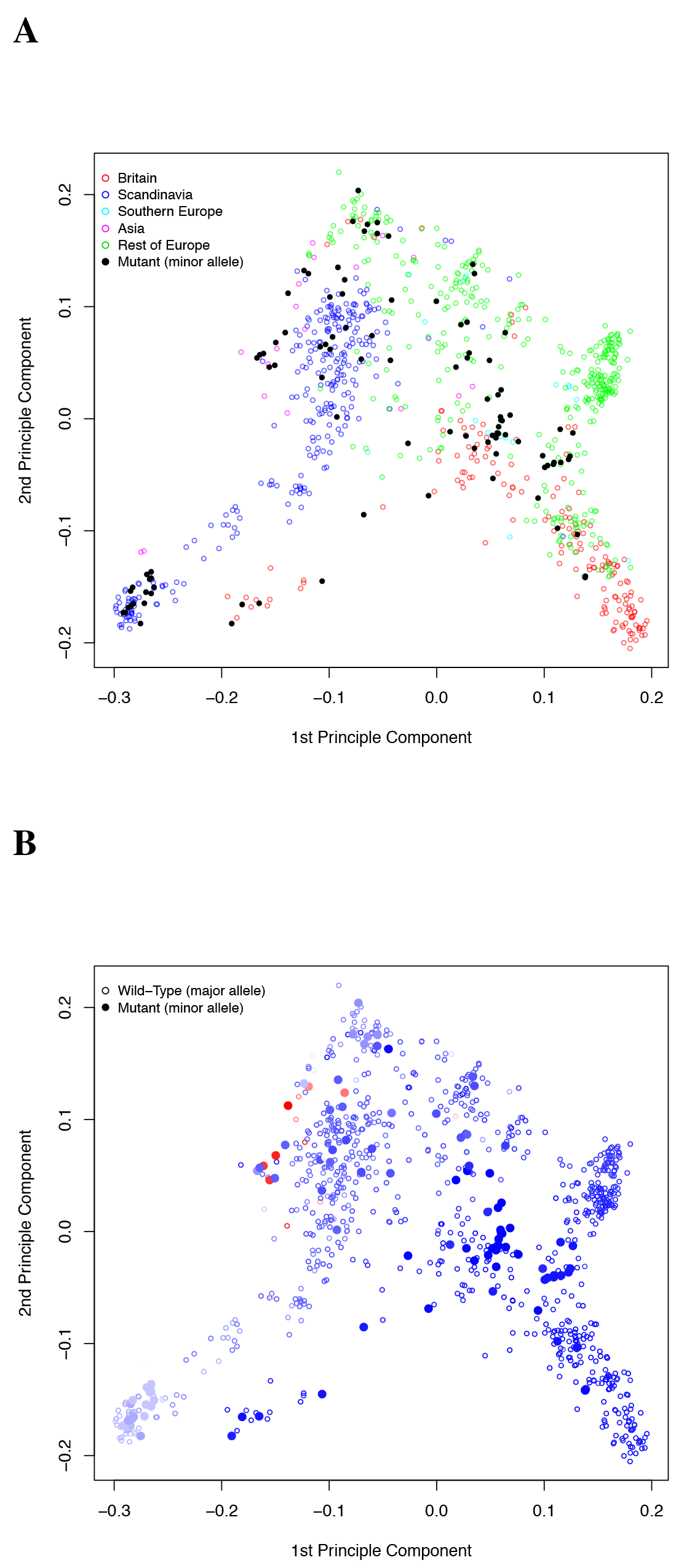

Supplement: S24 Figure — Principle components of the genomic kinship for the two alleles on chromosome 5 at 18,397,418 bp. Corresponding climate variable: number of consecutive cold days. A: Genomic kinship principle components categorized based on geographical regions. B: Genomic kinship principle components colored based on the scale of the climate variable. The colors scale from pure blue (the minimum climate variable value) to pure red (the maximum value). (TIF) [file pgen.1004842.s024.tif]

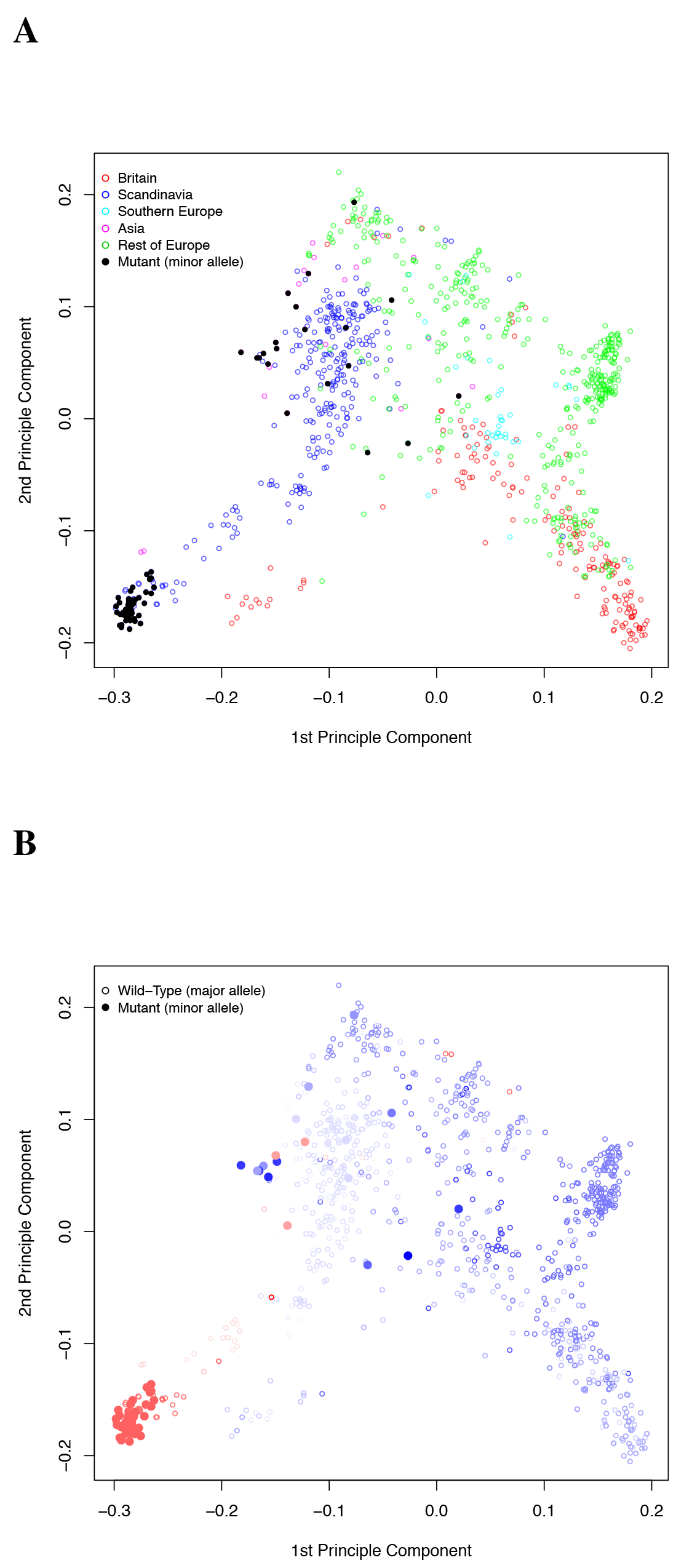

Supplement: S25 Figure — Principle components of the genomic kinship for the two alleles on chromosome 2 at 12,169,701 bp. Corresponding climate variable: day length in spring. A: Genomic kinship principle components categorized based on geographical regions. B: Genomic kinship principle components colored based on the scale of the climate variable. The colors scale from pure blue (the minimum climate variable value) to pure red (the maximum value). (TIF) [file pgen.1004842.s025.tif]

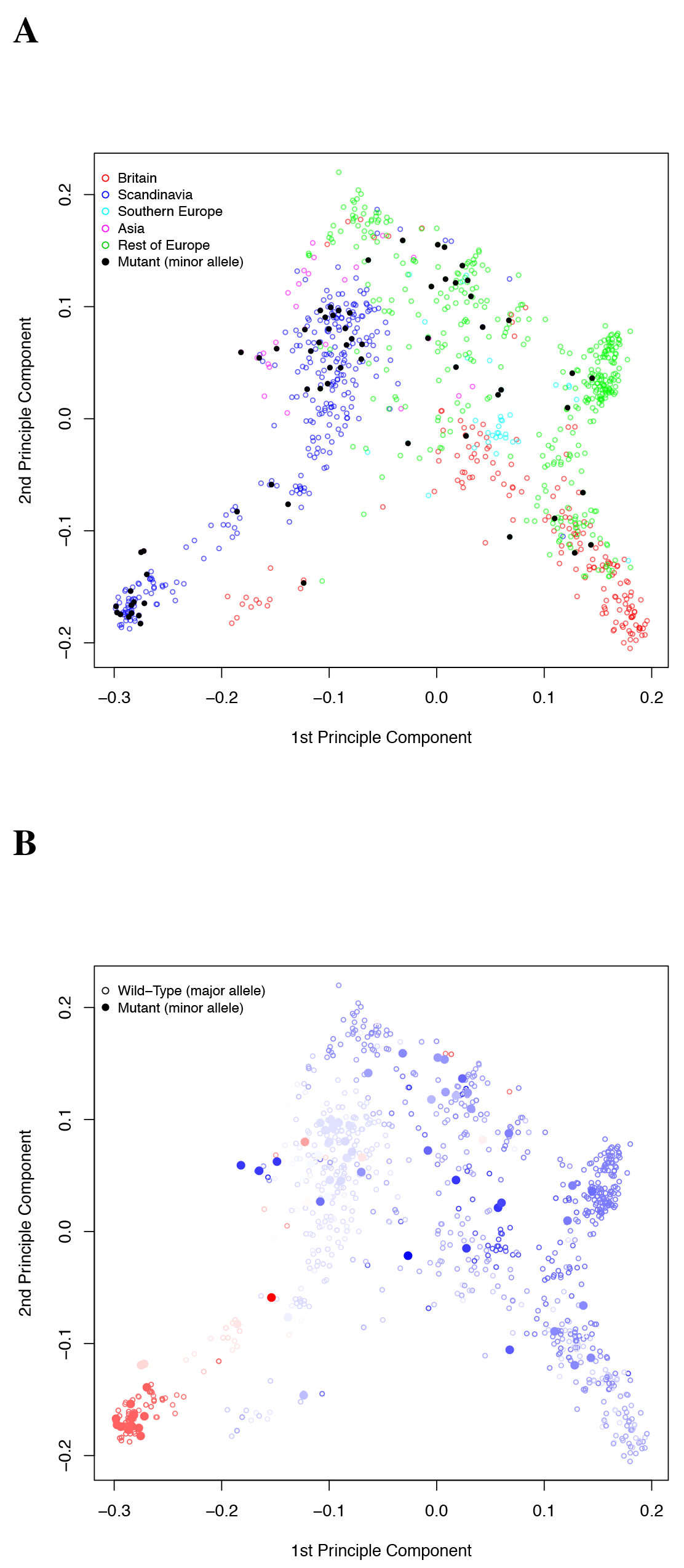

Supplement: S26 Figure — Principle components of the genomic kinship for the two alleles on chromosome 3 at 12,642,006 bp. Corresponding climate variable: day length in spring. A: Genomic kinship principle components categorized based on geographical regions. B: Genomic kinship principle components colored based on the scale of the climate variable. The colors scale from pure blue (the minimum climate variable value) to pure red (the maximum value). (TIF) [file pgen.1004842.s026.tif]

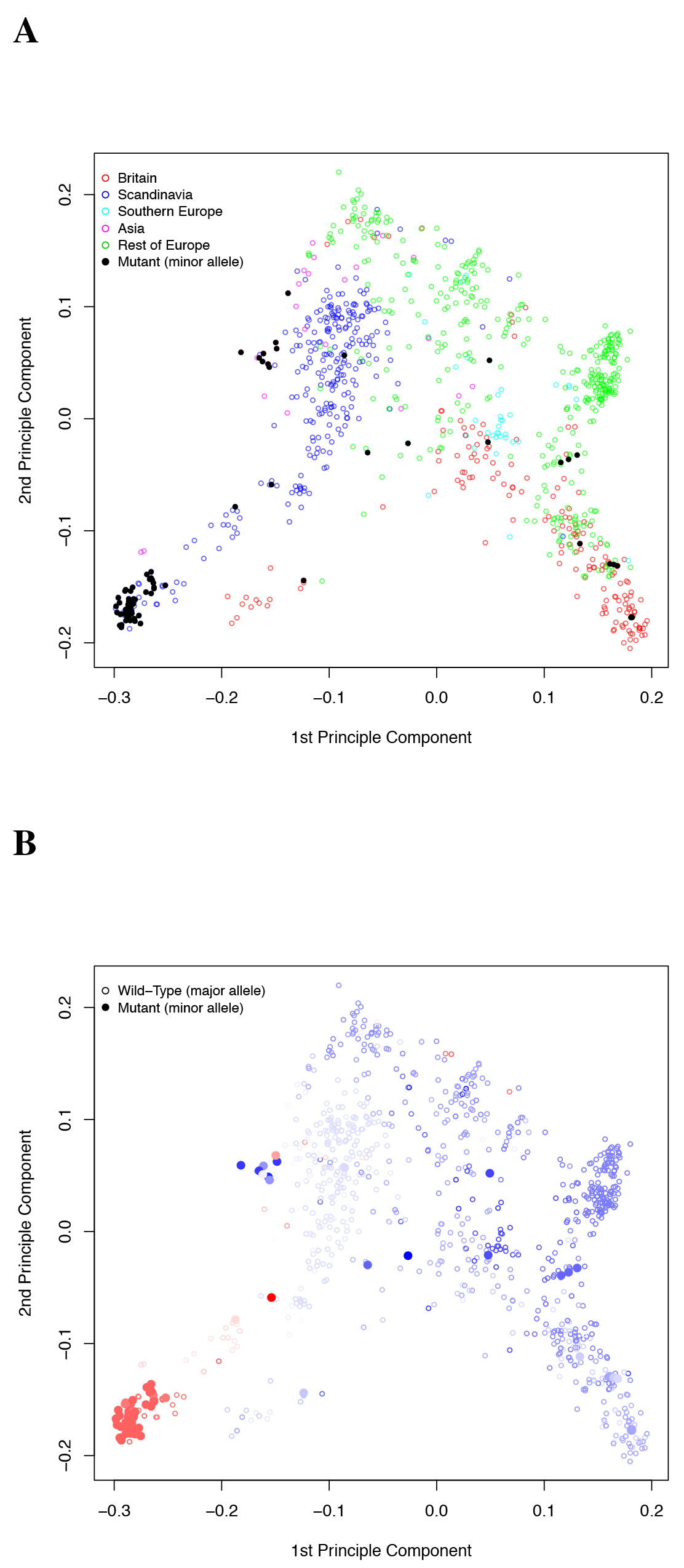

Supplement: S27 Figure — Principle components of the genomic kinship for the two alleles on chromosome 4 at 14,788,320 bp. Corresponding climate variable: day length in spring. A: Genomic kinship principle components categorized based on geographical regions. B: Genomic kinship principle components colored based on the scale of the climate variable. The colors scale from pure blue (the minimum climate variable value) to pure red (the maximum value). (TIF) [file pgen.1004842.s027.tif]

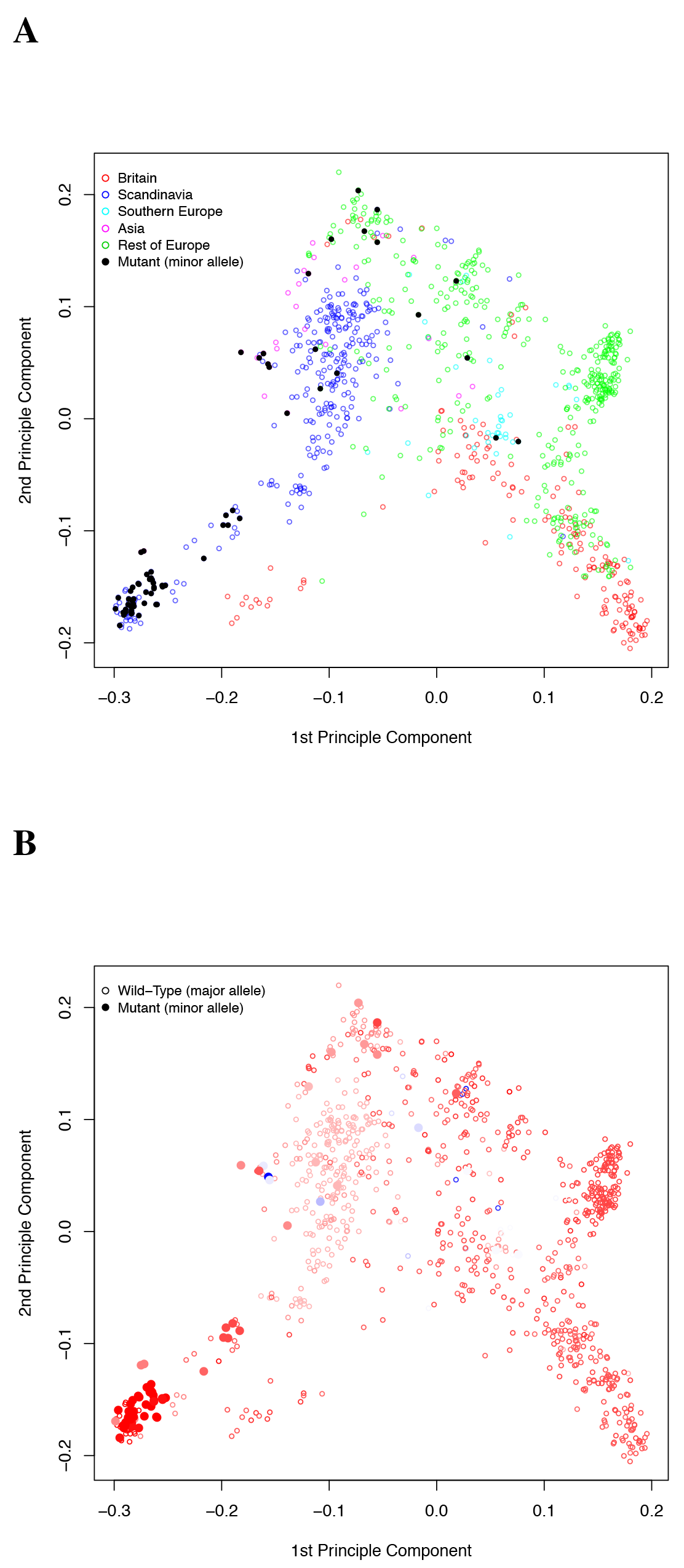

Supplement: S28 Figure — Principle components of the genomic kinship for the two alleles on chromosome 3 at 1,816,353 bp. Corresponding climate variable: relative humidity in spring. A: Genomic kinship principle components categorized based on geographical regions. B: Genomic kinship principle components colored based on the scale of the climate variable. The colors scale from pure blue (the minimum climate variable value) to pure red (the maximum value). (TIF) [file pgen.1004842.s028.tif]

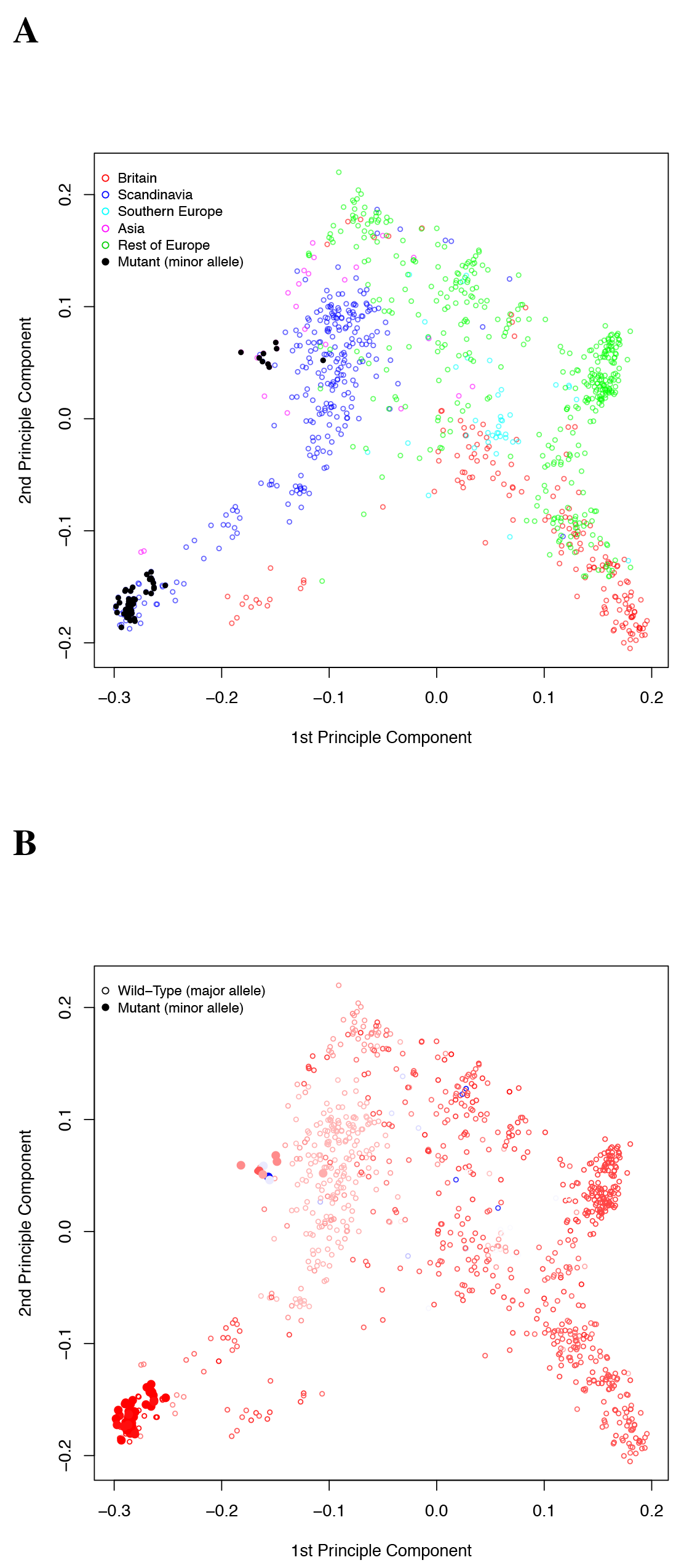

Supplement: S29 Figure — Principle components of the genomic kinship for the two alleles on chromosome 4 at 14,834,441 bp. Corresponding climate variable: relative humidity in spring. A: Genomic kinship principle components categorized based on geographical regions. B: Genomic kinship principle components colored based on the scale of the climate variable. The colors scale from pure blue (the minimum climate variable value) to pure red (the maximum value). (TIF) [file pgen.1004842.s029.tif]

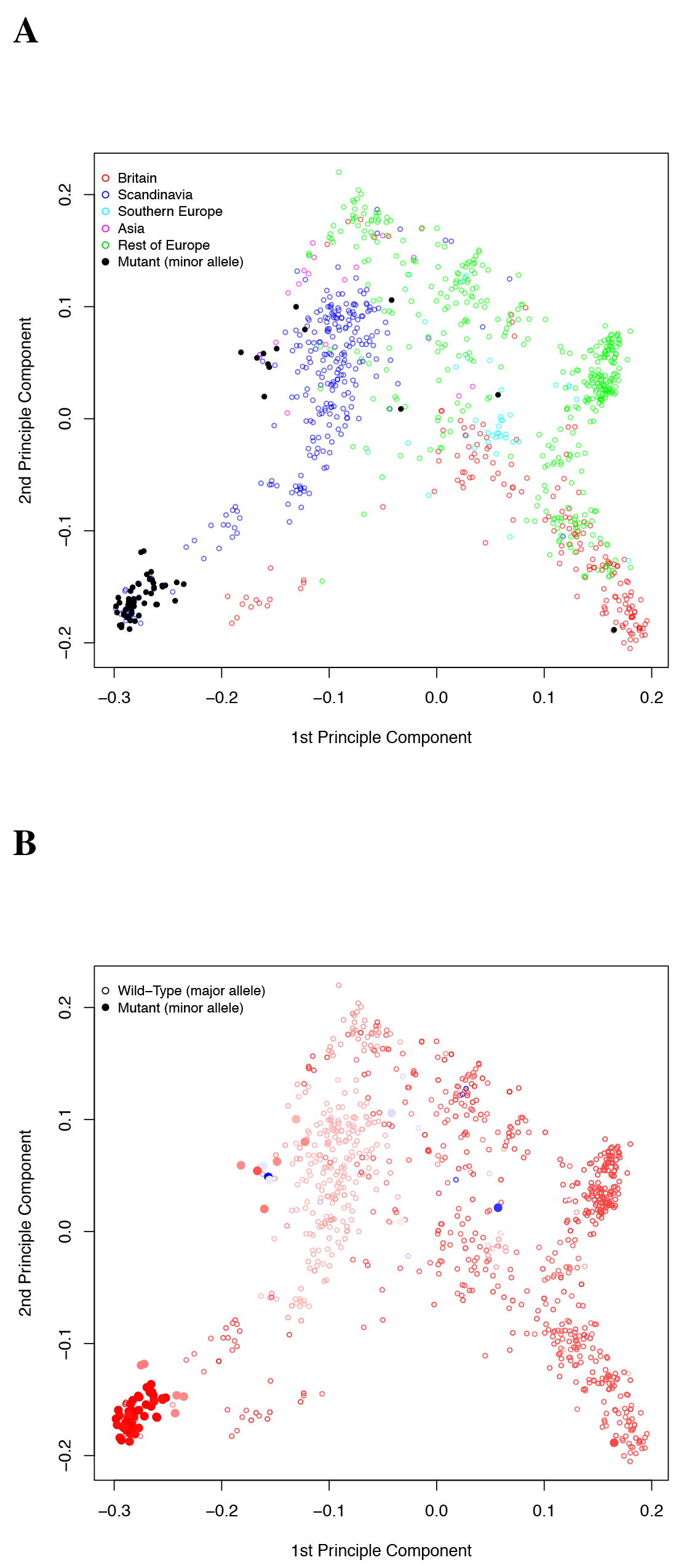

Supplement: S30 Figure — Principle components of the genomic kinship for the two alleles on chromosome 5 at 8,380,640 bp. Corresponding climate variable: relative humidity in spring. A: Genomic kinship principle components categorized based on geographical regions. B: Genomic kinship principle components colored based on the scale of the climate variable. The colors scale from pure blue (the minimum climate variable value) to pure red (the maximum value). (TIF) [file pgen.1004842.s030.tif]

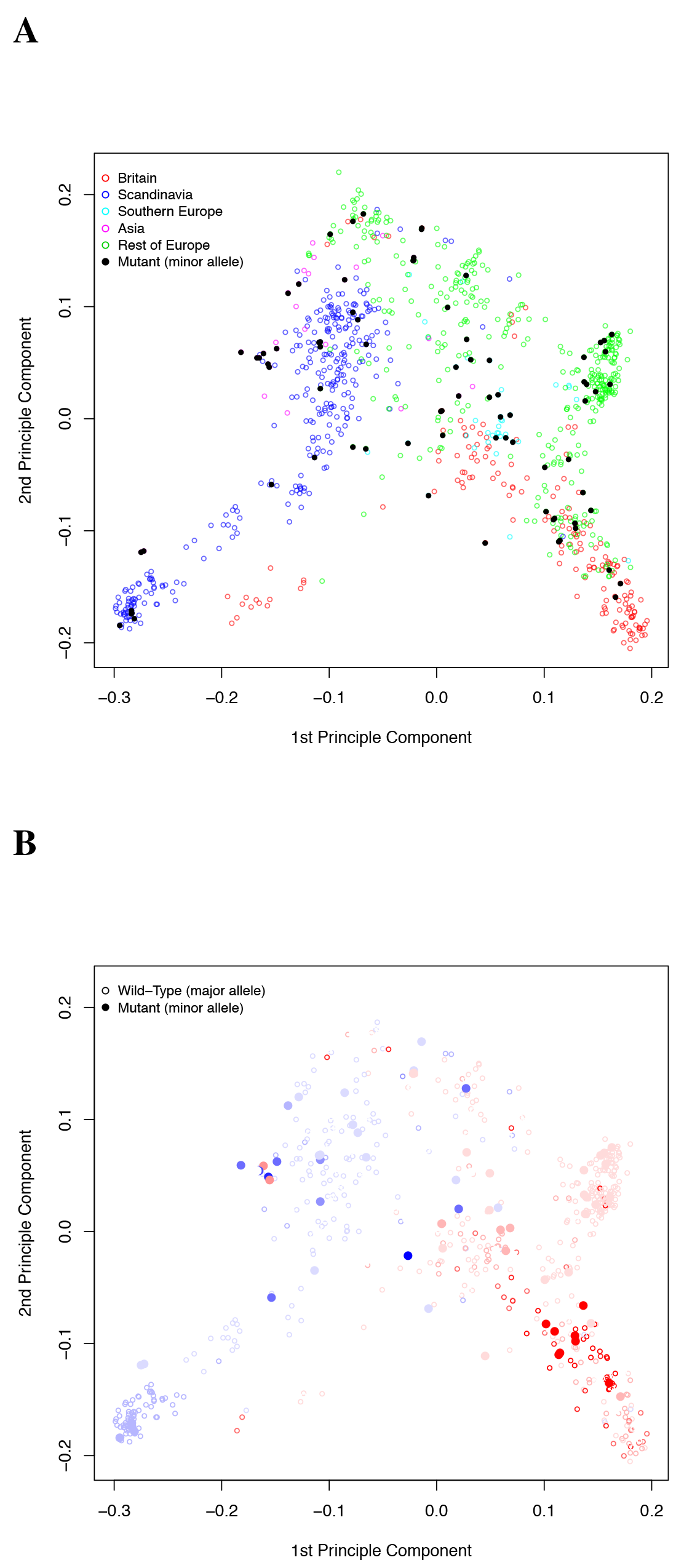

Supplement: S31 Figure — Principle components of the genomic kinship for the two alleles on chromosome 3 at 576,148 bp. Corresponding climate variable: length of the growing season. A: Genomic kinship principle components categorized based on geographical regions. B: Genomic kinship principle components colored based on the scale of the climate variable. The colors scale from pure blue (the minimum climate variable value) to pure red (the maximum value). (TIF) [file pgen.1004842.s031.tif]

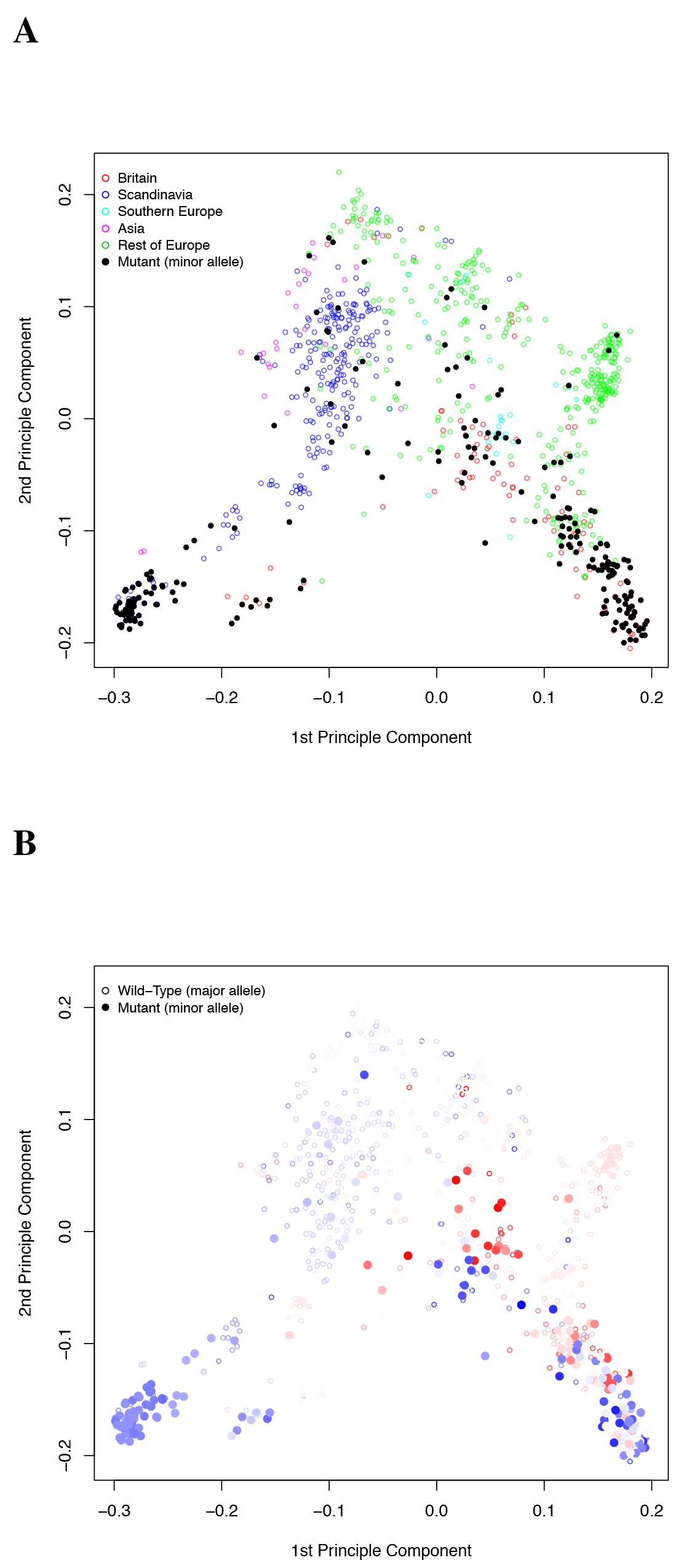

Supplement: S32 Figure — Principle components of the genomic kinship for the two alleles on chromosome 1 at 953,031 bp. Corresponding climate variable: number of consecutive frost-free days. A: Genomic kinship principle components categorized based on geographical regions. B: Genomic kinship principle components colored based on the scale of the climate variable. The colors scale from pure blue (the minimum climate variable value) to pure red (the maximum value). (TIF) [file pgen.1004842.s032.tif]

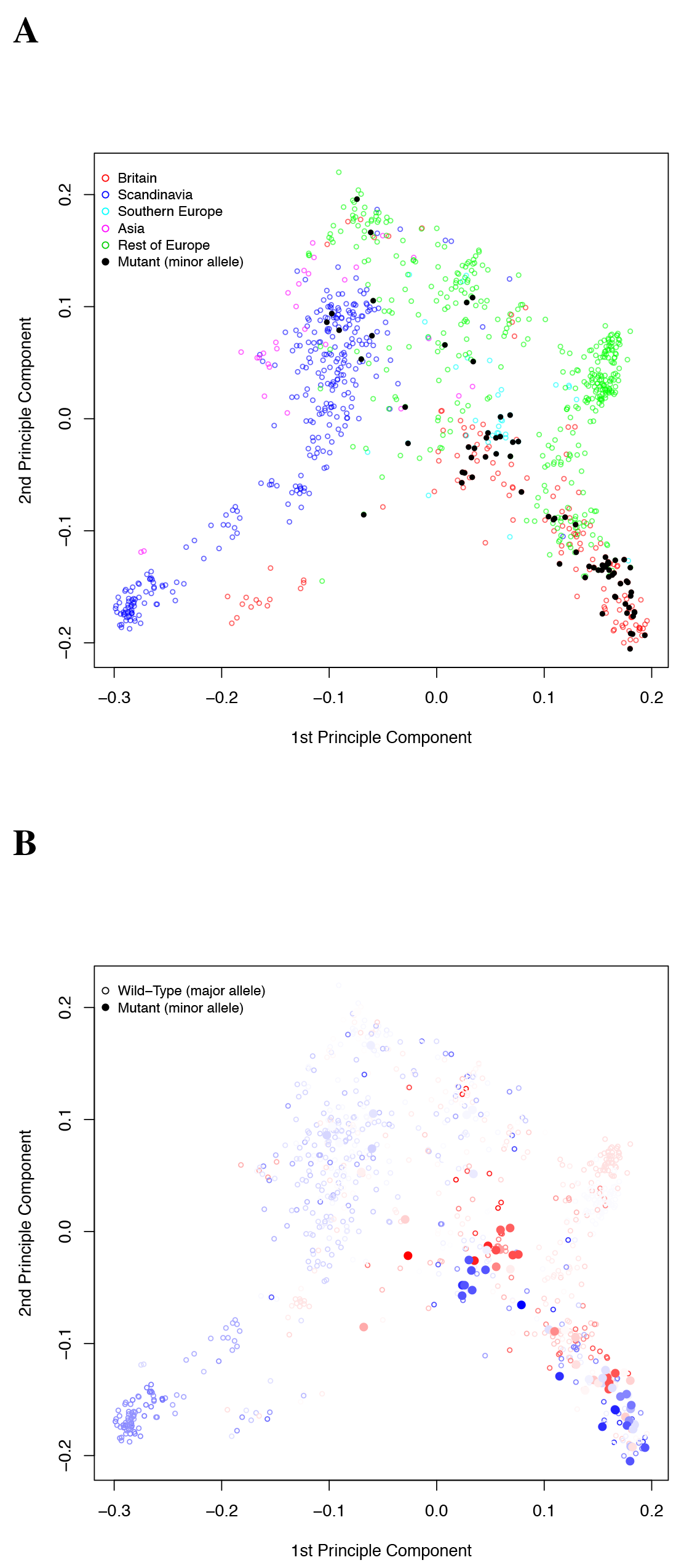

Supplement: S33 Figure — Principle components of the genomic kinship for the two alleles on chromosome 1 at 6,463,065 bp. Corresponding climate variable: number of consecutive frost-free days. A: Genomic kinship principle components categorized based on geographical regions. B: Genomic kinship principle components colored based on the scale of the climate variable. The colors scale from pure blue (the minimum climate variable value) to pure red (the maximum value). (TIF) [file pgen.1004842.s033.tif]

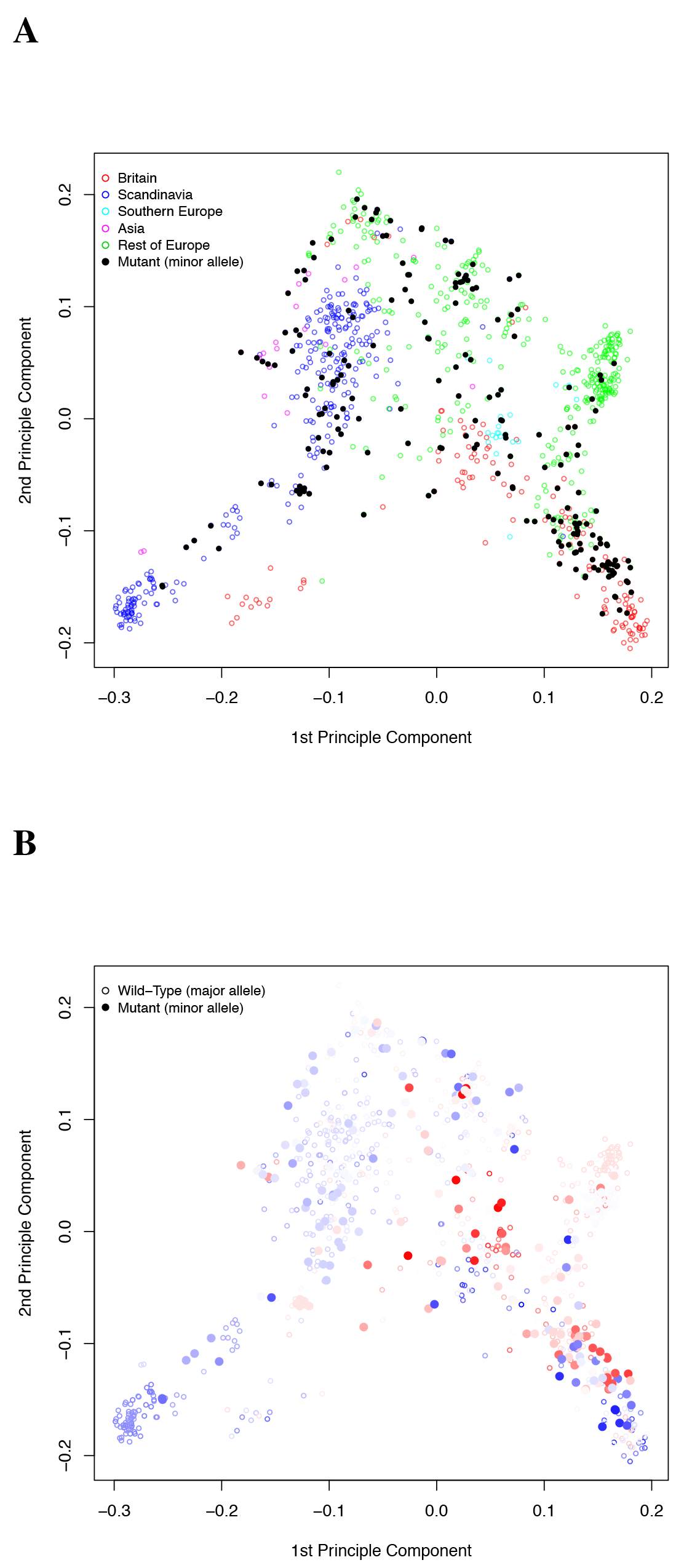

Supplement: S34 Figure — Principle components of the genomic kinship for the two alleles on chromosome 2 at 9,904,076 bp. Corresponding climate variable: number of consecutive frost-free days. A: Genomic kinship principle components categorized based on geographical regions. B: Genomic kinship principle components colored based on the scale of the climate variable. The colors scale from pure blue (the minimum climate variable value) to pure red (the maximum value). (TIF) [file pgen.1004842.s034.tif]

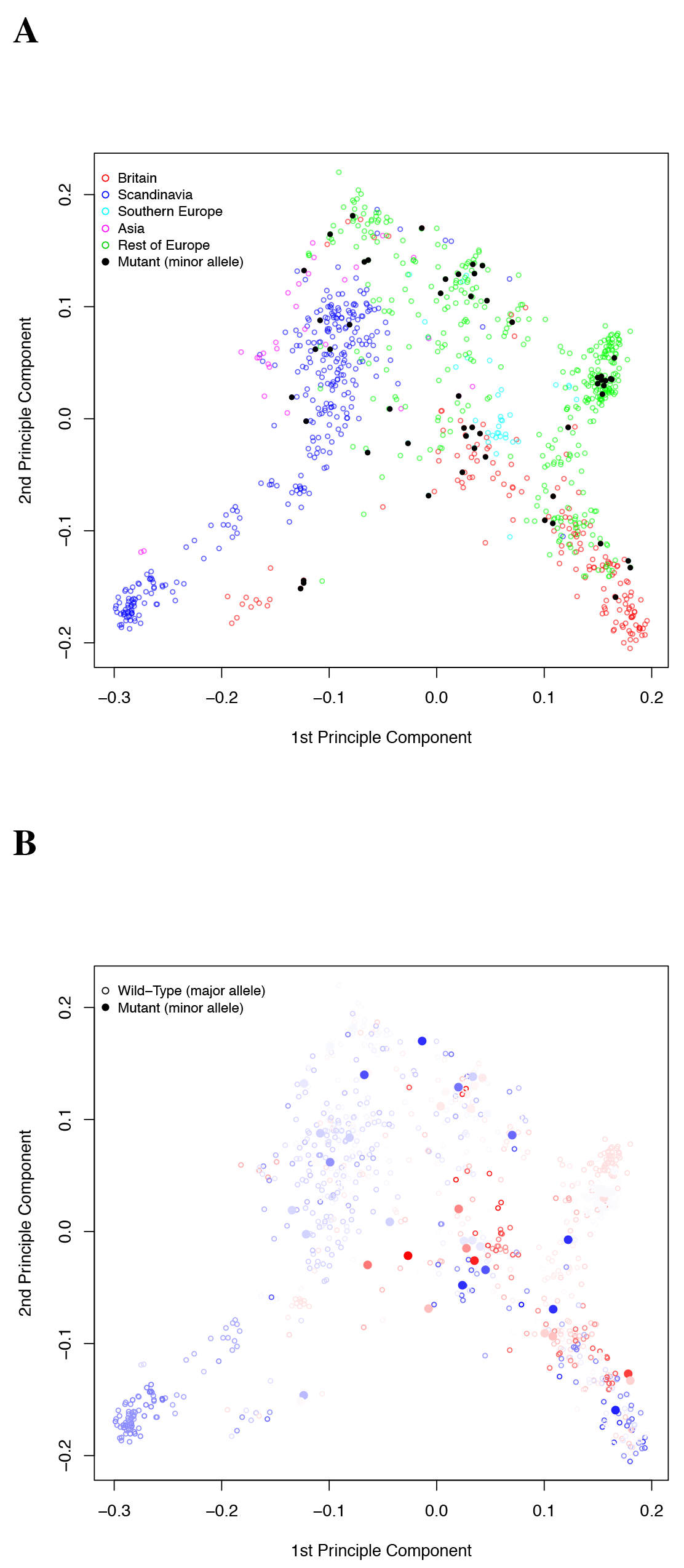

Supplement: S35 Figure — Principle components of the genomic kinship for the two alleles on chromosome 5 at 18,061,531 bp. Corresponding climate variable: number of consecutive frost-free days. A: Genomic kinship principle components categorized based on geographical regions. B: Genomic kinship principle components colored based on the scale of the climate variable. The colors scale from pure blue (the minimum climate variable value) to pure red (the maximum value). (TIF) [file pgen.1004842.s035.tif]

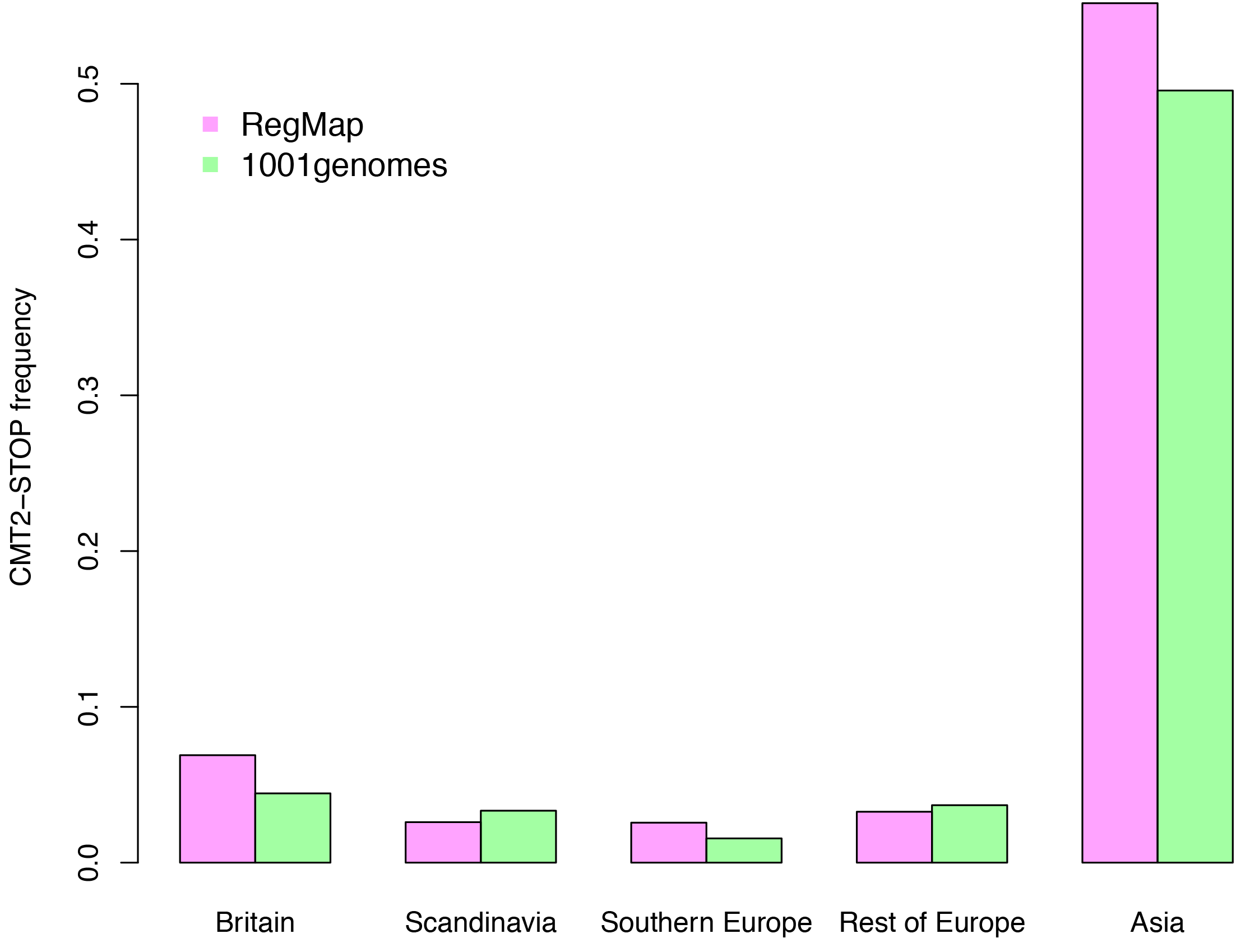

Supplement: S36 Figure — Comparison between the RegMap and 1001genomes collections in terms of the allele-frequency of CMT2STOP across different geographic regions in the Eurasian A. thaliana population. The numbers in the bars are the number of CMT2STOP alleles in this area. (TIF) [file pgen.1004842.s036.tif]

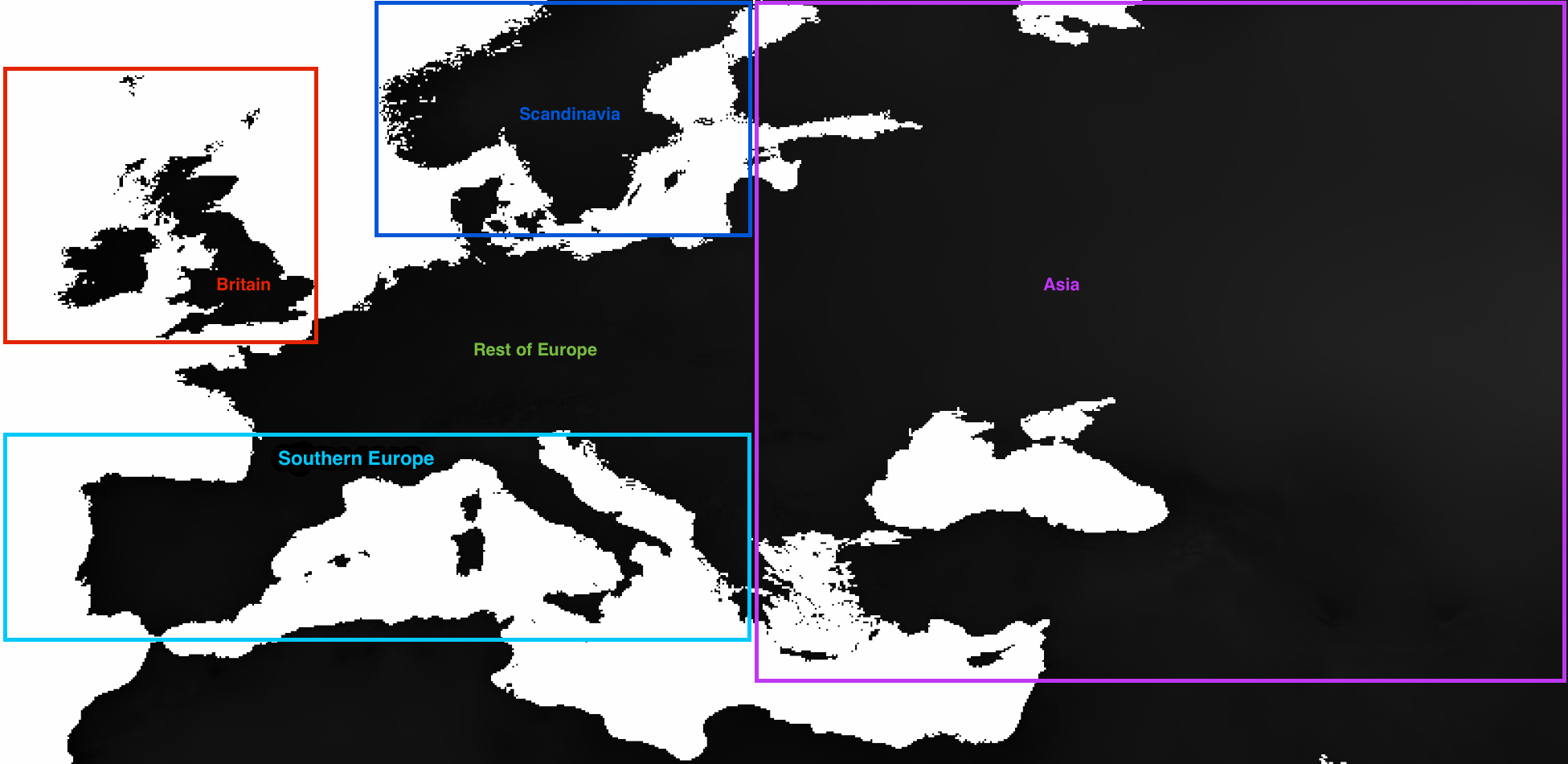

Supplement: S37 Figure — Defined geographical regions across the Eurasian sampling area. (TIF) [file pgen.1004842.s037.tif]

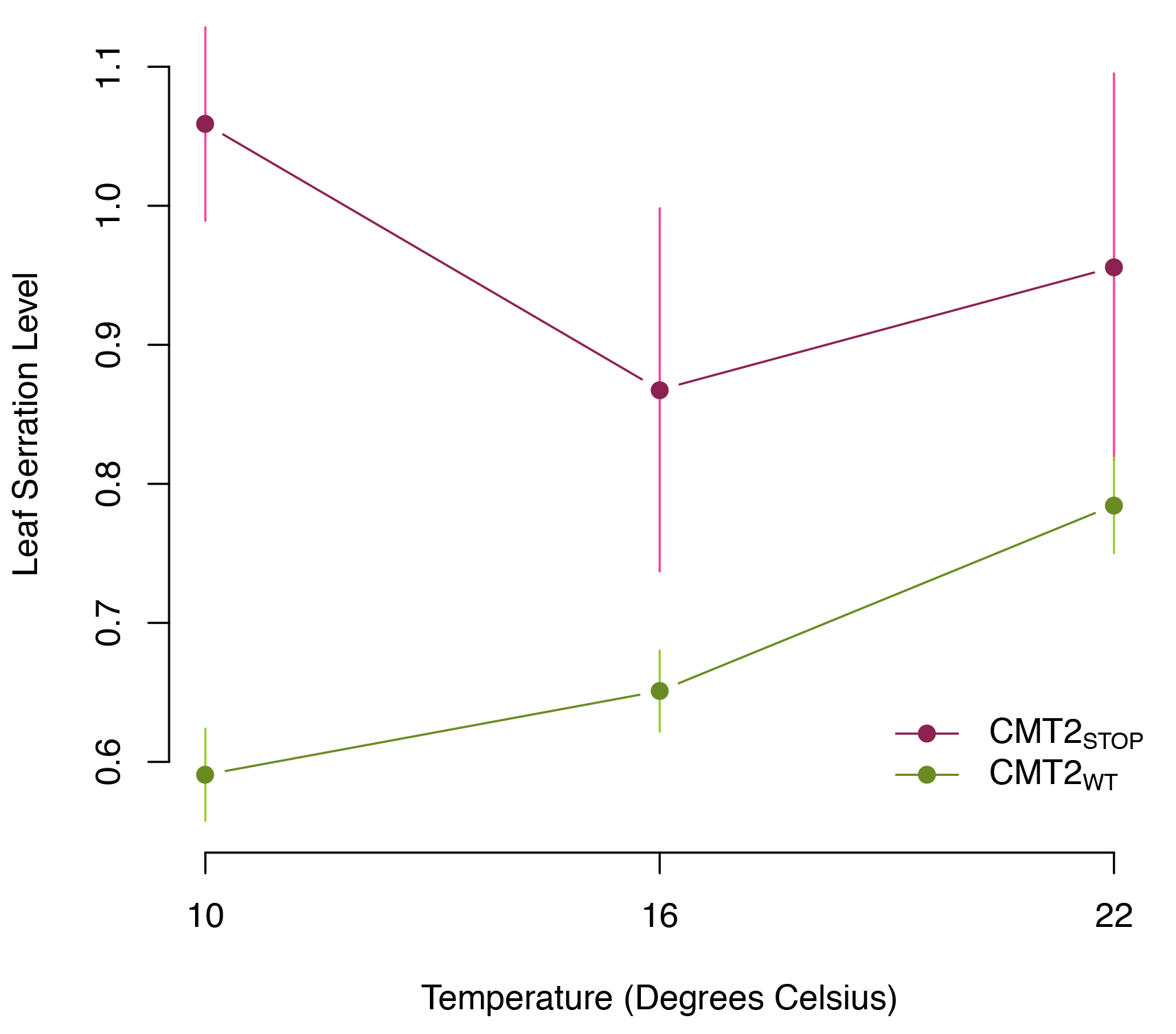

Supplement: S38 Figure — CMT2-by-temperature interaction effects on leaf serration. The analysis was performed using the genome-wide association data reported by [28]. Each point is the mean leaf serration level of a combination of CMT2 genotype and temperature. The vertical bars represent standard errors of the mean estimates. (TIF) [file pgen.1004842.s038.tif]

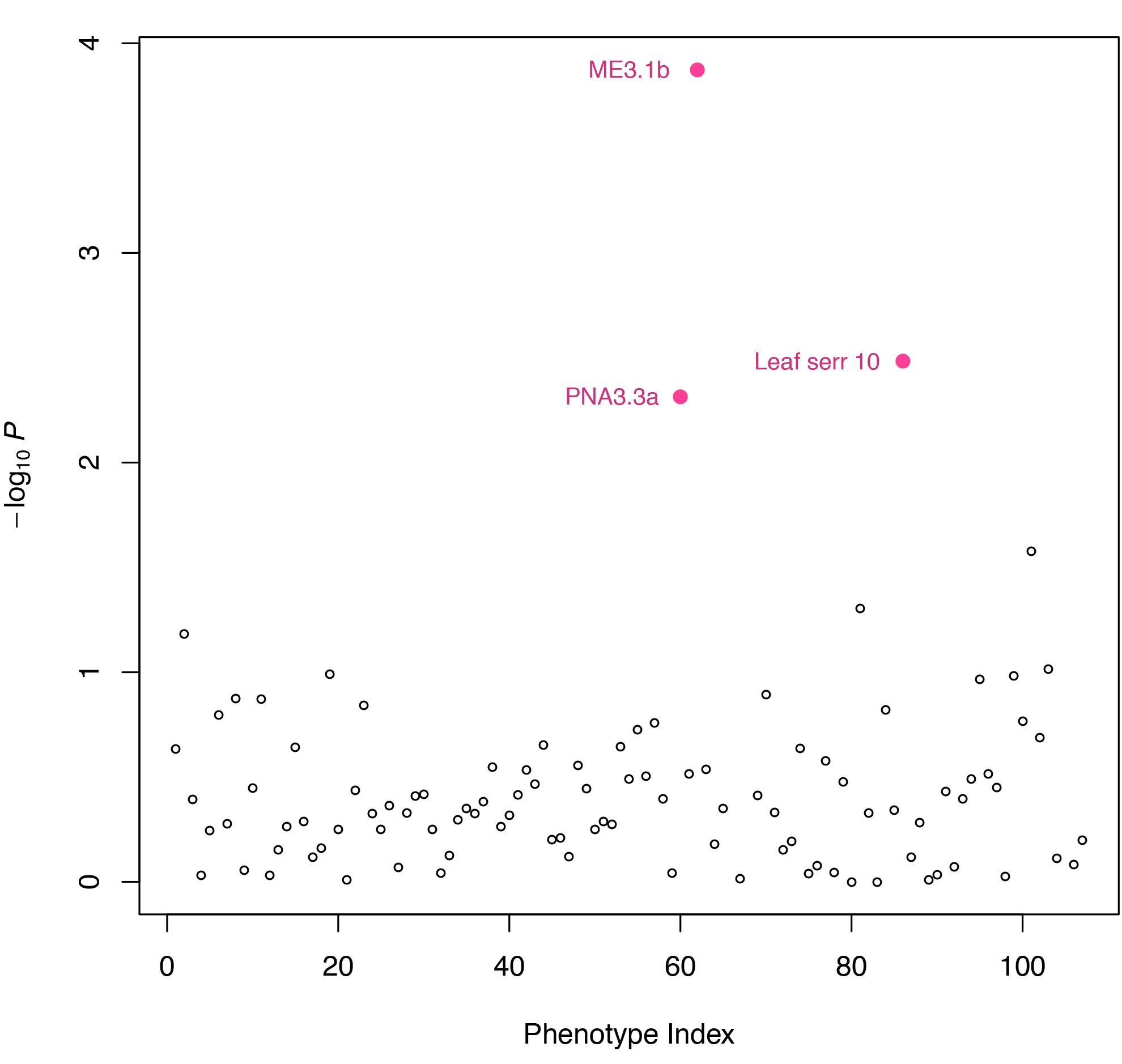

Supplement: S39 Figure — Associations between the CMT2STOP genotype and the 107 scored phenotypes in [28]. The most significant three associations are labeled in pink, with a false discovery rate of 0.17. The definition of each labeled phenotype should be referred to the Tables in [28]. (TIF) [file pgen.1004842.s039.tif]

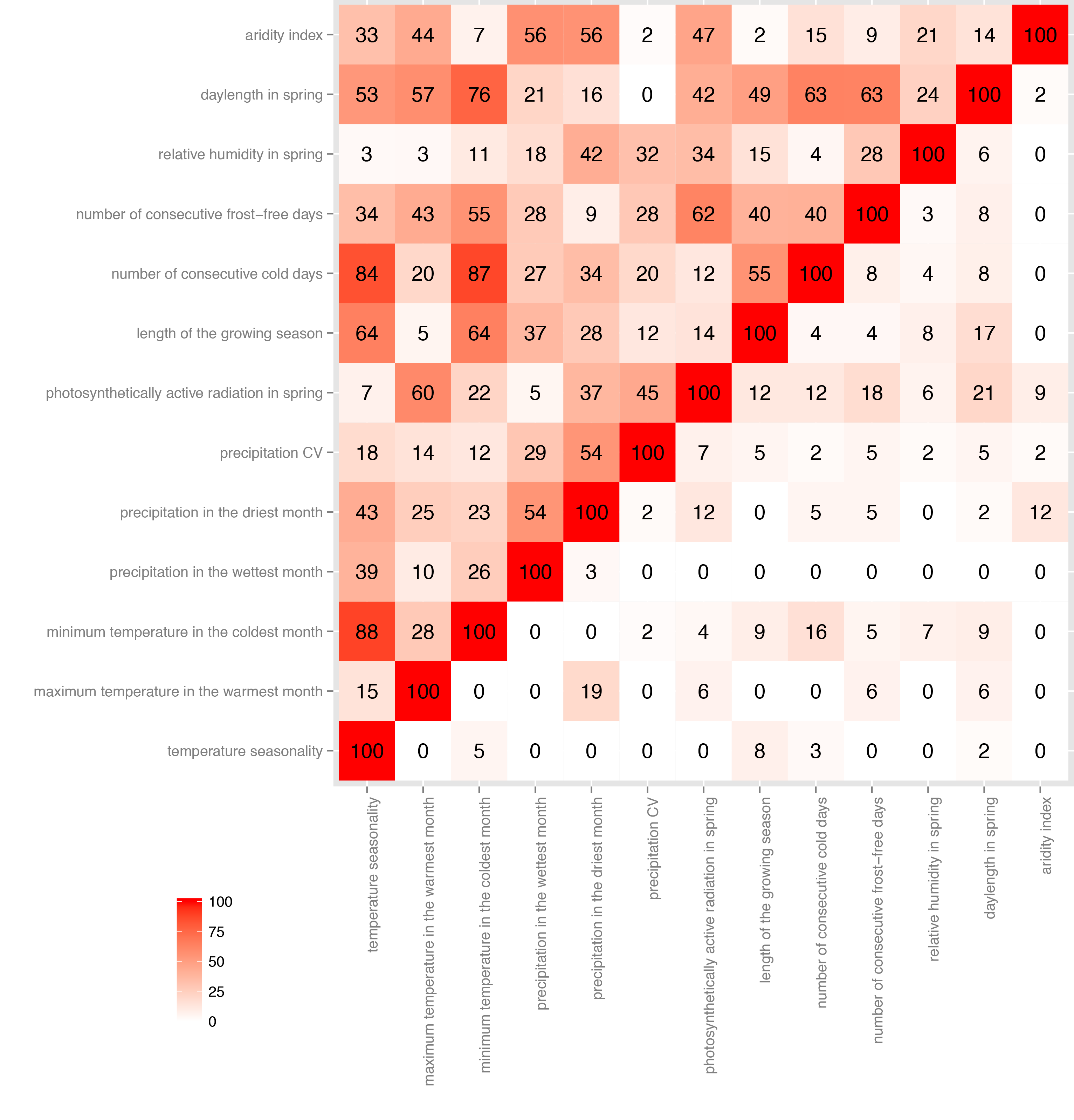

Supplement: S40 Figure — Comparison between the correlations among the climate variables (upper triangle) and the overlap in variance-heterogeneity GWA profiles (lower triangle). Numbers shown in the figure are percentages. Pearson's correlation coefficients were calculated for each pair of the climate variables. Overlaps in GWA profiles were calculated as the proportion of shared SNPs above the threshold of 1.0×10^−4^. (TIF) [file pgen.1004842.s040.tif]

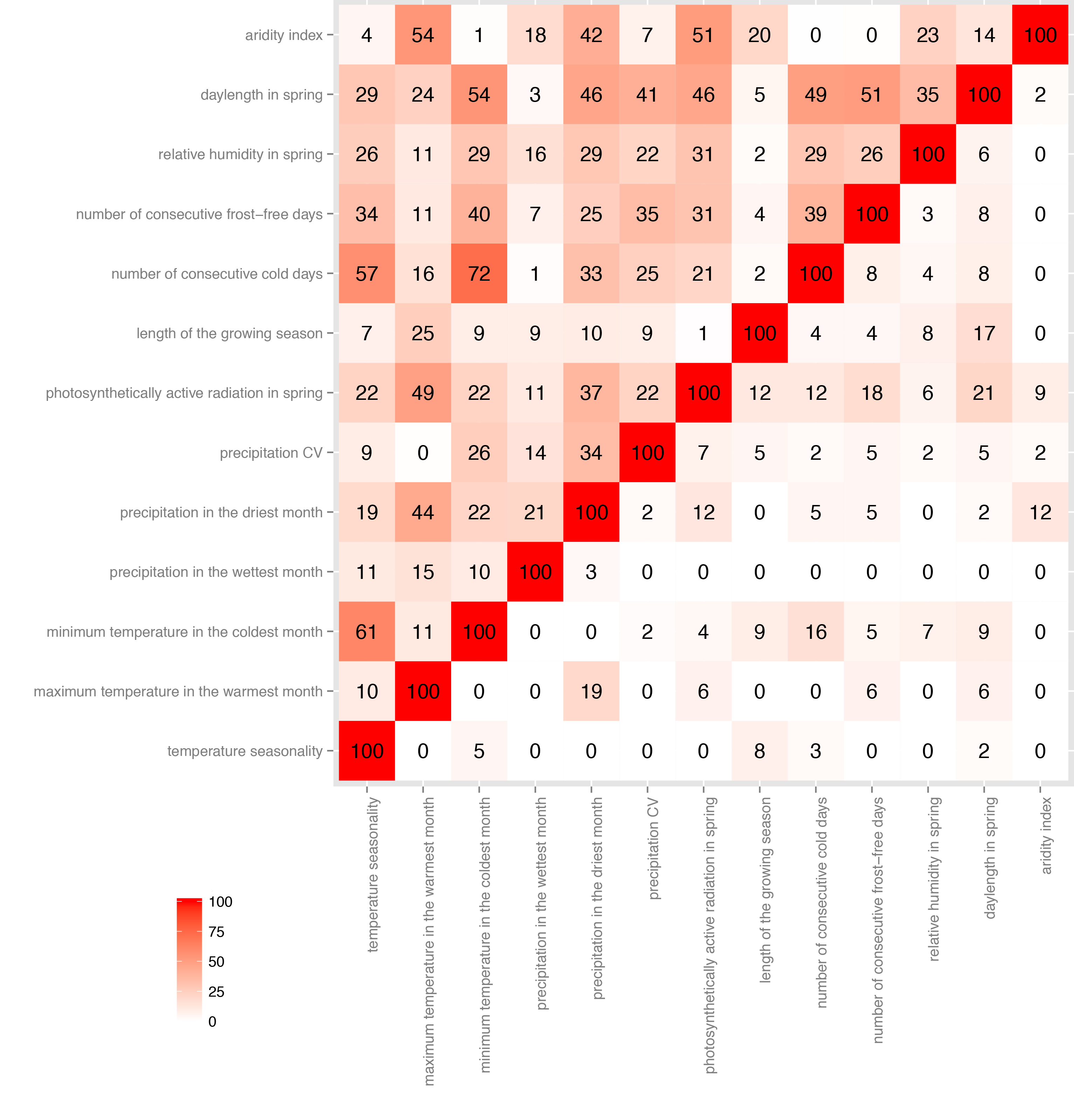

Supplement: S41 Figure — Comparison between the correlations among the residual climate variables after genomic kinship correction (upper triangle) and the overlap in variance-heterogeneity GWA profiles (lower triangle). Numbers shown in the figure are percentages. Pearson's correlation coefficients were calculated for each pair of the climate variables. Overlaps in GWA profiles were calculated as the proportion of shared SNPs above the threshold of 1.0×10^−4^. (TIF) [file pgen.1004842.s041.tif]

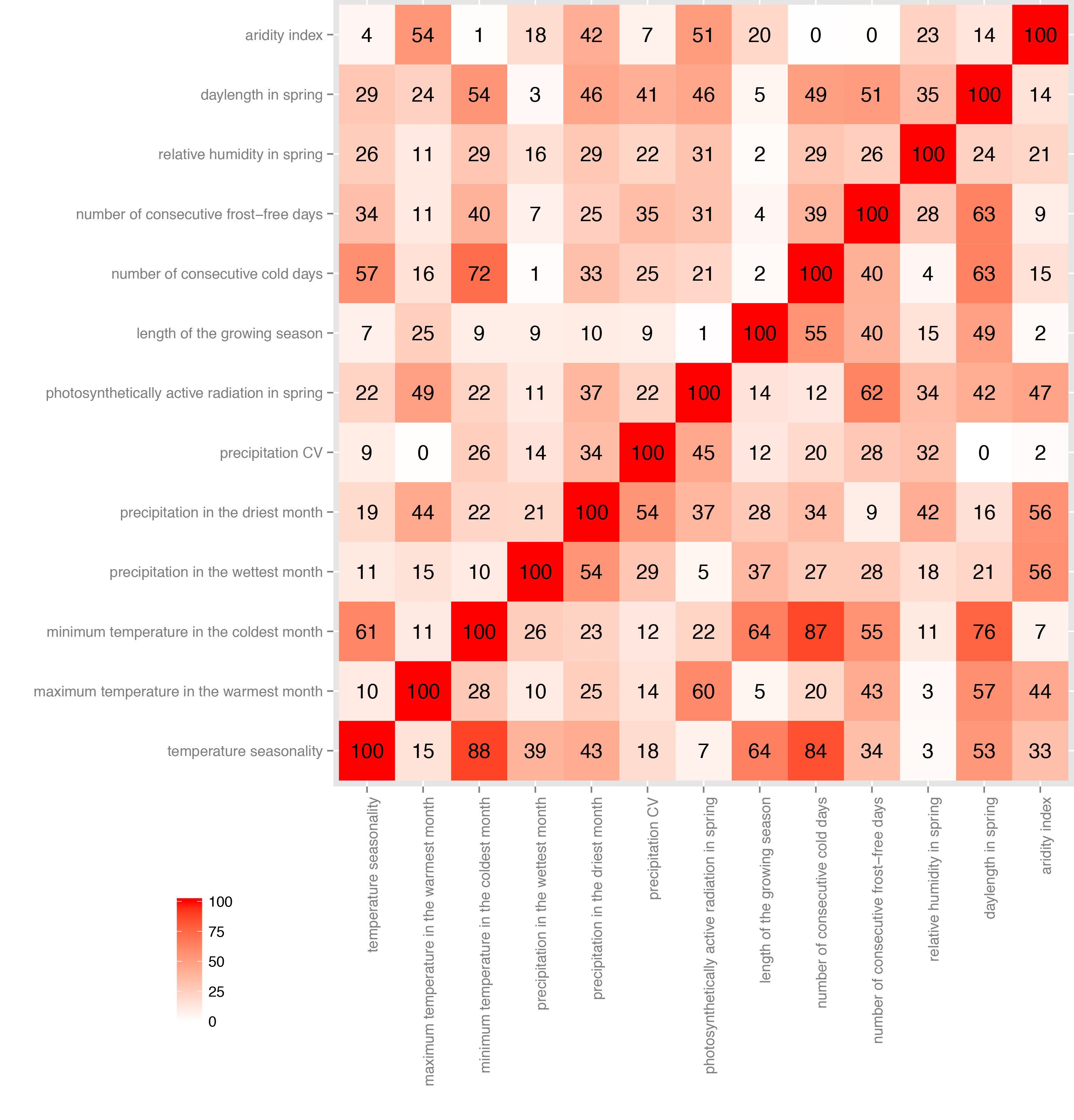

Supplement: S42 Figure — Comparison between the correlations among the residual climate variables after genomic kinship correction (upper triangle) and the correlations among the original climate variables (lower triangle). Numbers shown in the figure are percentages. Pearson's correlation coefficients were calculated for each pair of the climate variables. (TIF) [file pgen.1004842.s042.tif]

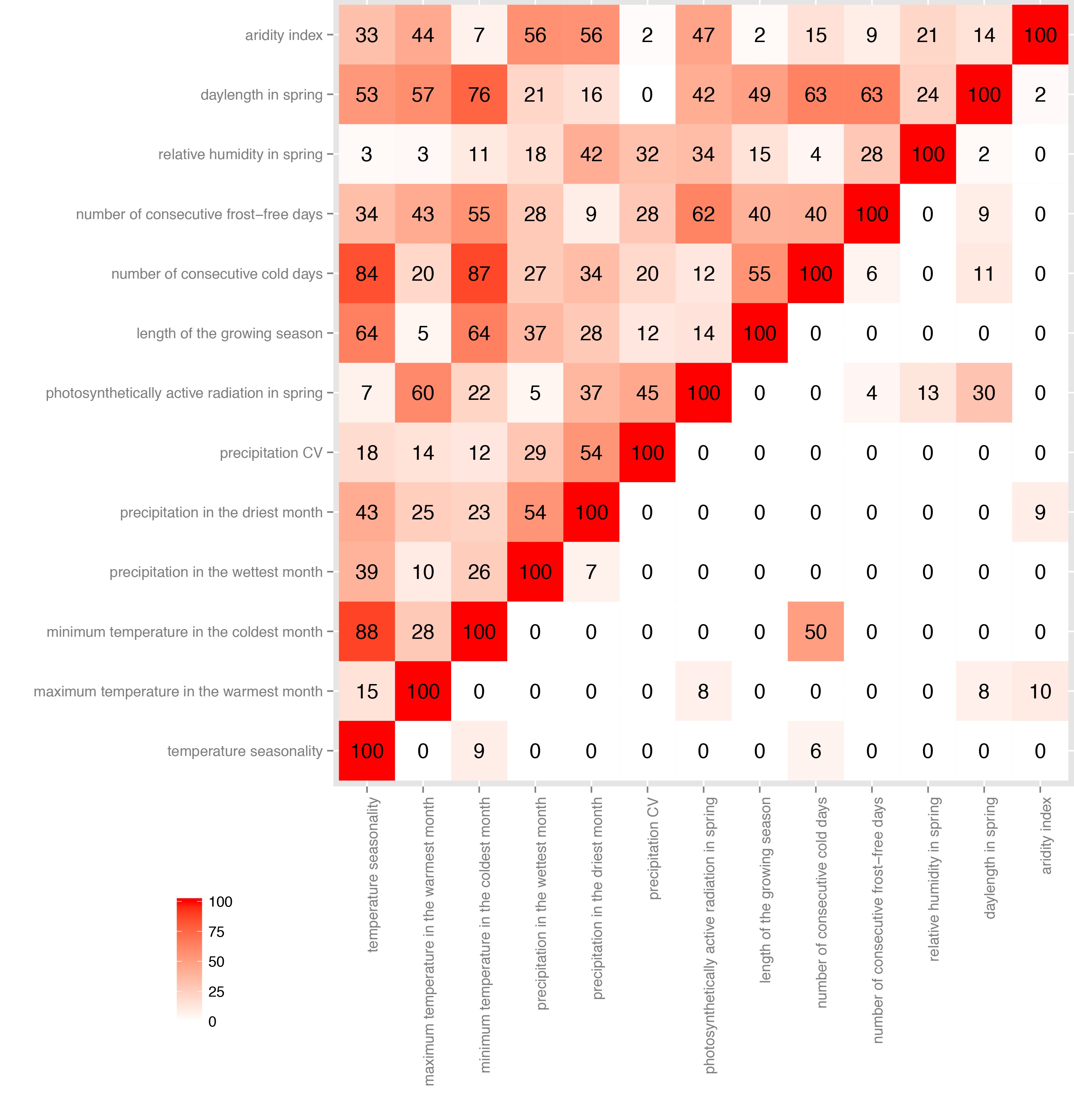

Supplement: S43 Figure — Comparison between the correlations among the climate variables (upper triangle) and the overlap in ordinary GWA profiles (lower triangle). Numbers shown in the figure are percentages. Pearson's correlation coefficients were calculated for each pair of the climate variables. Overlaps in GWA profiles were calculated as the proportion of shared SNPs above the threshold of 1.0×10^−4^. (TIF) [file pgen.1004842.s043.tif]

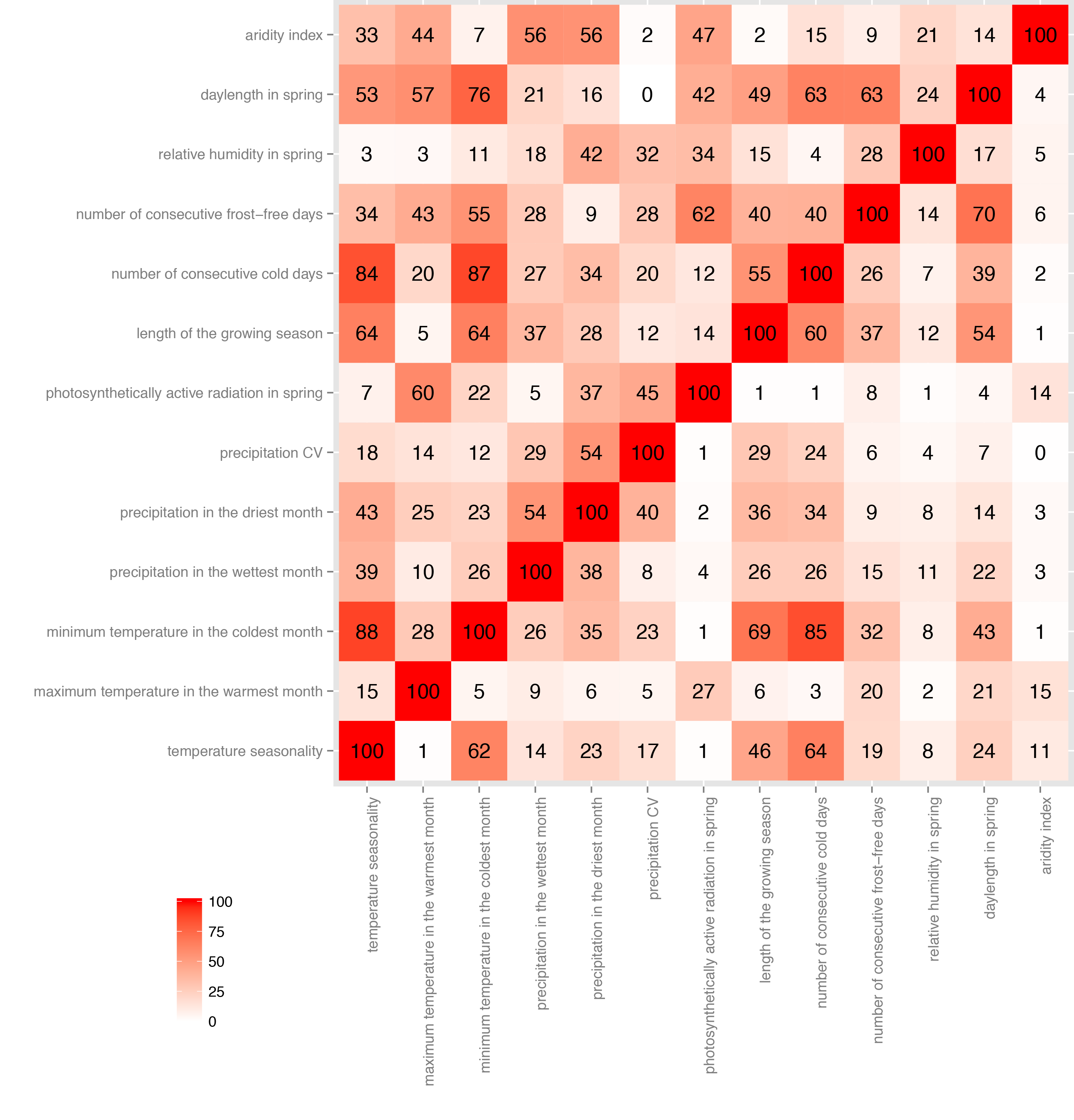

Supplement: S44 Figure — Comparison between the correlations among the climate variables (upper triangle) and the overlap in simple GWA profiles without correction for population structure (lower triangle). Numbers shown in the figure are percentages. Pearson's correlation coefficients were calculated for each pair of the climate variables. Overlaps in GWA profiles were calculated as the proportion of shared SNPs above the threshold of 1.0×10^−4^. (TIF) [file pgen.1004842.s044.tif]

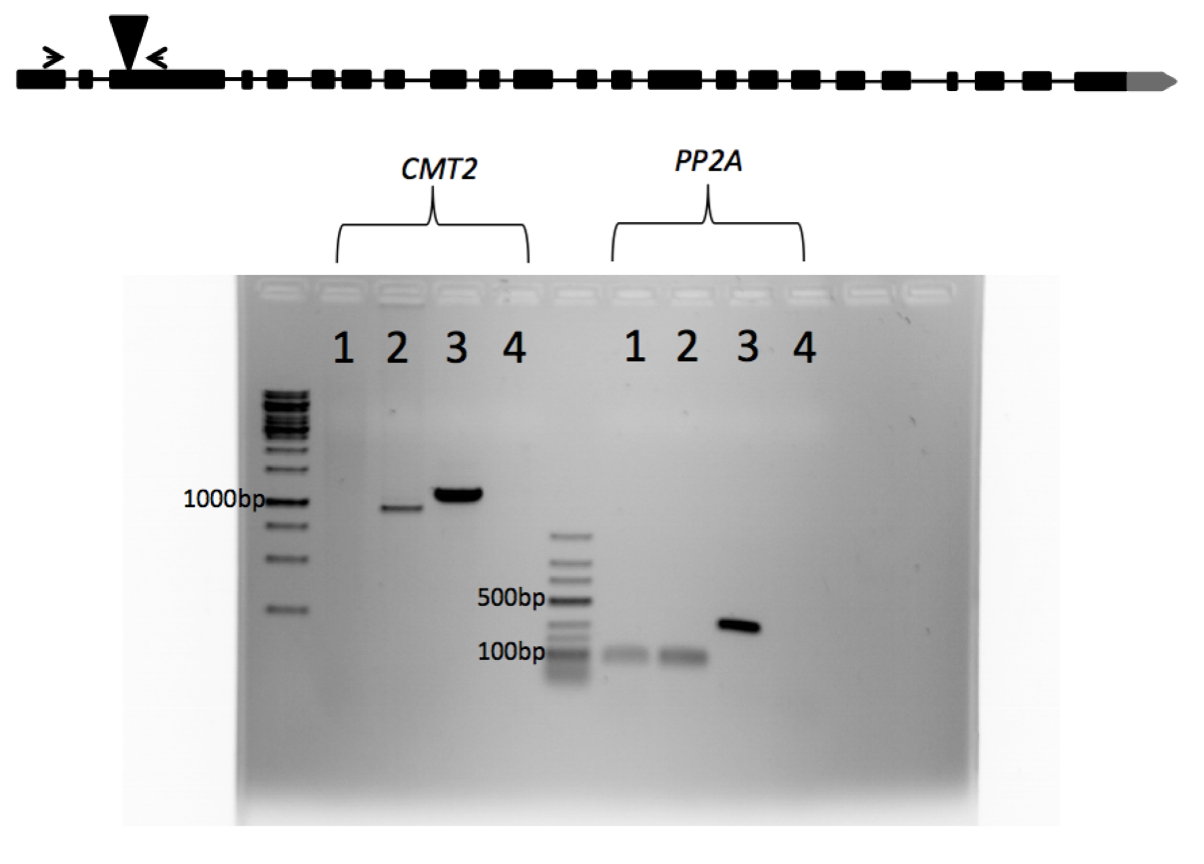

Supplement: S45 Figure — Gene-model of CMT2 and T-DNA insertion confirmation. Boxes indicate exons, lines represent introns. The triangle shows the T-DNA insertion site. Arrow heads indicate the location of primers that were used to assay CMT2 transcripts. CMT2: PCR reaction with CMT2-specific primers, PP2A: PCR reaction with PP2A-specific primers. Lanes 1: cmt2-5 cDNA, lanes 2: Col cDNA, lanes 3: Col genomic DNA, lanes 4: no template controls. CMT2 cDNA and genomic DNA are predicted to give 940bp and 1159bp bands, respectively. PP2A cDNA and genomic DNA are predicted to give 84 bp and 210 bp bands, respectively. (TIF) [file pgen.1004842.s045.tif]

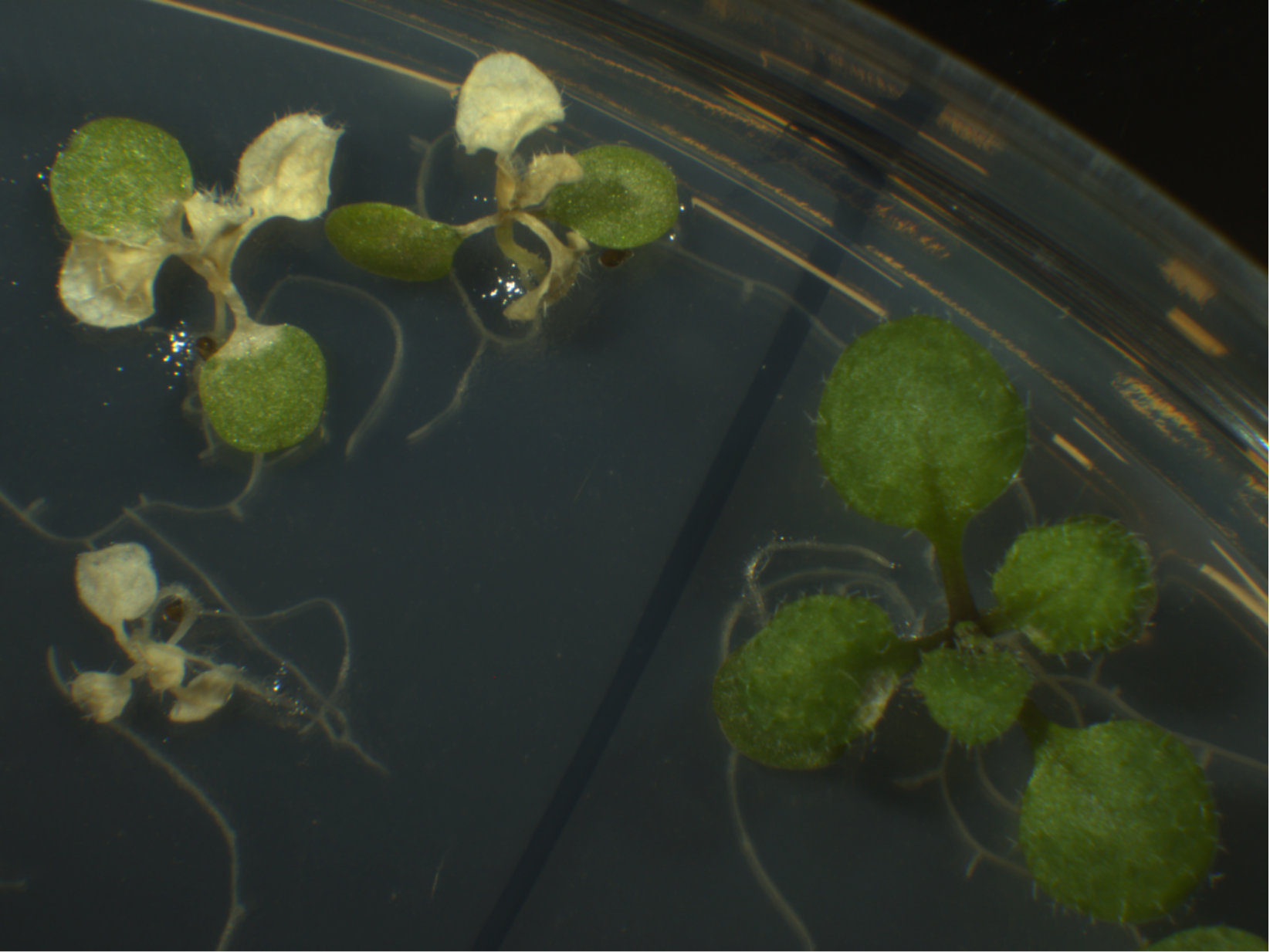

Supplement: S46 Figure — Prolonged heat stress is often lethal. Ten-day-old seedlings were heat-stressed at 37.5°C for 24 h based on a published protocol [27]. Plants were counted as non-viable if shoot apices were completely bleached. Note that the lamina of cotyledons often remains green for a longer time but no recovery was observed if apices were bleached. (TIF) [file pgen.1004842.s046.tif]
