# Supplementary material for: Natural CMT2 Variation Is Associated With Genome-Wide Methylation Changes and Temperature Seasonality
Source: PLoS Genet. 2014 Dec 11;10(12):e1004842. doi: 10.1371/journal.pgen.1004842 (PMC4263395; doi:10.1371/journal.pgen.1004842)
Supplement: S1 Table — Detailed information about the missense mutations significantly associated with climate adaptability of Arabidopsis thaliana. (PDF) [file pgen.1004842.s047.pdf]

Table S1: Mis- and non-sense mutations in high-LD with genome-wide significant, non-additive associations to climate adaptability.

Trait	Chrom	Pos (bp)	Locus	Gene name	Consequence	Protein position	AA Change	Codon change	MAF	Mutant analysis	
										PASE	MSA
Temperature seasonality											
	4	10 405 599	AT4G19000	IWS2	missense	33	S/T	Tcg/Acg	0.11	0.21	0.35
	4	10 410 039	AT4G19006		missense	240	H/L	cAc/cTc	0.11	0.52	0.41
	4	10 411 801	AT4G19010		missense	539	N/H	Aac/Cac	0.11	0.29	0.08
	4	10 412 877	AT4G19010		missense	373	S/L	tCg/tTg	0.11	0.52	0.14
	4	10 413 290	AT4G19010		missense	311	A/V	gCa/gTa	0.12	0.35	0.68
	4	10 413 374	AT4G19010		missense	283	K/R	aAg/aGg	0.12	0.23	0.2
	4	10 414 556	AT4G19020	CMT2	stop	11	E/*	Gag/Tag	0.11	STOP	STOP
	4	10 414 640	AT4G19020	CMT2	frameshift	39	-	-	0.00	FS	FS
	4	10 414 640	AT4G19020	CMT2	missense	39	E/K	Gaa/Aaa	0.12	0.63	0.17
	4	10 414 747	AT4G19020	CMT2	missense	74	N/K	aaC/aaG	0.11	0.45	0.11
	4	10 415 805	AT4G19020	CMT2	missense	354	G/D	gGc/gAc	0.11	0.58	0.11
	4	10 415 833	AT4G19020	CMT2	missense	363	L/F	ttA/ttT	0.11	0.2	0.14
	4	10 416 047	AT4G19020	CMT2	missense	435	G/S	Ggt/Agt	0.11	0.26	0.14
	4	10 445 425	AT4G19060		missense	270	I/T	aTt/aCt	0.11	0.37	0.44
	4	10 447 160	AT4G19070		missense	140	S/R	Agt/Cgt	0.11	0.73	0.11
	4	10 450 001	AT4G19090		missense	17	R/G	Aga/Gga	0.11	0.93	0.07
	4	10 455 784	AT4G19110		missense	192	L/F	ttG/ttT	0.10	0.2	0.45
	4	10 455 784	AT4G19110		missense	248	L/F	ttG/ttT	0.10	0.2	0.44
	4	10 455 784	AT4G19110		missense	248	L/F	ttG/ttT	0.10	0.2	0.44
Maximum temperature in the warmest month											
	1	6 936 457	AT1G19990		missense	183	S/F	tCt/tTt	0.09	0.64	0.2
Minimum temperature in the coldest month											
	5	14 067 526	AT5G35930		missense	963	E/Q	Gaa/Caa	0.12	0.3	0.05
Number of consecutive cold days											
	5	7 492 033	AT5G22560		missense	355	E/D	gaG/gaC	0.14	0.26	0.22
	5	7 492 259	AT5G22560		missense	280	R/T	aGa/aCa	0.13	0.63	0.11
	5	7 492 277	AT5G22560		missense	274	N/T	aAt/aCt	0.14	0.27	0.11
	5	7 492 277	AT5G22560		missense	274	N/S	aAt/aGt	0.14	0.3	0.11
Relative humidity in spring & Day length in spring											
	4	14 788 320	AT4G30200	VEL1	missense	236	E/D	gaA/gaT	0.11	0.26	0.06
	4	14 788 320	AT4G30200	VEL1	missense	253	E/D	gaA/gaT	0.11	0.26	0.06
	4	14 829 581	AT4G30290	XTH19	missense	100	I/V	Att/Gtt	0.07	0.14	0.49
Minimum temperature in the coldest month & Number of consecutive cold days											
	2	19 397 389	AT2G47240		missense	620	K/R	aAa/aGa	0.06	0.23	0.13
	2	19 397 389	AT2G47240		missense	620	K/R	aAa/aGa	0.06	0.23	0.13
Temperature seasonality & Day length in spring											
	2	12 169 734	AT2G28470	BGAL8	missense	672	E/D	gaA/gaT	0.11	0.26	0.06
	2	12 169 734	AT2G28470	BGAL8	missense	678	E/D	gaA/gaT	0.11	0.26	0.06
	2	12 169 828	AT2G28470	BGAL8	missense	647	F/Y	tTc/tAc	0.12	0.36	0.72
	2	12 169 828	AT2G28470	BGAL8	missense	641	F/Y	tTc/tAc	0.12	0.36	0.72
Number of consecutive frost-free days											
	1	954 782	AT1G03790	SOM	missense	65	N/I	aAt/aTt	0.25	0.6	0.06
	1	955 189	AT1G03790	SOM	missense	201	P/T	Cct/Act	0.25	0.22	0.17
	1	955 268	AT1G03790	SOM	missense	227	S/C	tCt/tGt	0.25	0.55	0.4
