# Supplementary material for: Natural CMT2 Variation Is Associated With Genome-Wide Methylation Changes and Temperature Seasonality
Source: PLoS Genet. 2014 Dec 11;10(12):e1004842. doi: 10.1371/journal.pgen.1004842 (PMC4263395; doi:10.1371/journal.pgen.1004842)
Supplement: S2 Table — Loci significantly associated with climate adaptability of Arabidopsis thaliana but without non-synonymous mutations in high LD detected. P-values were obtained from linear regression of squared z-scores. GC P-values were the P-values after genomic control. Gamma P-values were obtained by fitting generalized linear models with Gamma response. Pleiotropic loci are marked with stars. bp = base pair; MAF = minor allele frequency. (PDF) [file pgen.1004842.s048.pdf]

Table S2: Genes located less than 100Kb up- or down-stream of the leading SNP in the Genome-Wide Association analysis and that also are in high linkage disequilibrium with the SNP ($r^2 > 0.8$)

Trait	Gene	
Temperature seasonality		
	AT4G18960	AG K-box region and MADS-box transcription factor family protein
	AT4G18970	GDSL-like Lipase/Acylhydrolase superfamily protein
	AT4G18975	Pentatricopeptide repeat (PPR) superfamily protein
	AT4G18980	AtS40-3
	AT4G18990	XTH29 xyloglucan endotransglucosylase/hydrolase 29
	AT4G19003	VPS25 E2F/DP family winged-helix DNA-binding domain
	AT4G19030	NLM1 NOD26-like major intrinsic protein 1
	AT4G19035	LCR7 low-molecular-weight cysteine-rich 7
	AT4G19038	LCR15 low-molecular-weight cysteine-rich 15
	AT4G19040	EDR2 ENHANCED DISEASE RESISTANCE 2
	AT4G19045	Mob1/phocein family protein
	AT4G19050	NB-ARC domain-containing disease resistance protein
	AT4G19080	unknown protein
	AT4G19095	unknown protein
	AT4G19100	unknown protein
	AT4G19112	CPuORF25 conserved peptide upstream open reading frame 25
	AT4G19120	ERD3 S-adenosyl-L-methionine-dependent methyltransferases superfamily protein
Maximum temperature in the warmest month		
	AT1G19970	ER lumen protein retaining receptor family protein
	AT1G19980	cytomatrix protein-related
	AT1G20000	TAF11b TBP-associated factor 11B
	AT1G20010	TUB5 tubulin beta-5 chain
	AT1G20015	snoRNA
Minimum temperature in the coldest month		
	AT5G35926	Protein with RNI-like/FBD-like domains
Number of consecutive cold days		
	AT5G22555	unknown protein
	AT5G22570	WRKY38 WRKY DNA-binding protein 38
Day length in spring		
	AT3G30859	transposable element gene
	AT3G30867	pseudogene, putative SNF8 protein homolog
Relative humidity in spring & Day length in spring		
	AT4G30240	Syntaxin/t-SNARE family protein
	AT4G30250	P-loop containing nucleoside triphosphate hydrolases superfamily protein
	AT4G30260	Integral membrane Yip1 family protein
	AT4G30270	MERI5B xyloglucan endotransglucosylase/hydrolase 24
	AT4G30280	ATXTH18 xyloglucan endotransglucosylase/hydrolase 18
	AT4G30300	ATNAP15 non-intrinsic ABC protein 15
	AT4G30320	CAP (Cysteine-rich secretory proteins, Antigen 5, and Pathogenesis-related 1 protein) superfamily protein
	AT4G30330	Small nuclear ribonucleoprotein family protein
	AT4G30340	ATDGK7 diacylglycerol kinase 7
Minimum temperature in the coldest month & Number of consecutive cold days		
	AT2G47250	RNA helicase family protein
	AT2G45150	CDS4 cytidinediphosphate diacylglycerol synthase 4
	AT2G45160	HAM1 GRAS family transcription factor
	AT2G45161	unknown protein

	AT2G45170	ATATG8E AUTOPHAGY 8E
	AT5G45380	ATDUR3 solute:sodium symporters;urea transmembrane transporters
	AT5G45390	CLPP4 CLP protease P4
	AT5G45400	RPA70C Replication factor-A protein 1-related
	AT5G45410	unknown protein
Temperature seasonality & Day length in spring		
	AT2G28410	unknown protein
	AT2G28420	Lactoylglutathione lyase / glyoxalase I family protein
	AT2G28426	unknown protein
	AT2G28430	unknown protein
	AT2G28440	proline-rich family protein
	AT2G28450	zinc finger (CCCH-type) family protein
	AT2G28460	Cysteine/Histidine-rich C1 domain family protein
Relative humidity in spring		
	AT3G06019	unknown protein
	AT3G06020	unknown protein
	AT3G06030	ANP3 NPK1-related protein kinase 3
	AT5G24530	DMR6 2-oxoglutarate (2OG) and Fe(II)-dependent oxygenase superfamily protein
	AT5G24540	BGLU31 beta glucosidase 31
Length of the growing season		
	AT3G02660	Tyrosyl-tRNA synthetase, class Ib, bacterial/mitochondrial
	AT3G02670	Glycine-rich protein family
	AT3G02680	NBS1 nijmegen breakage syndrome 1
	AT3G02690	nodulin MtN21 /EamA-like transporter family protein
Number of consecutive frost-free days		
	AT1G03780	TPX2 targeting protein for XKLP2
	AT1G03800	ERF10 ERF domain protein 10
	AT1G03810	Nucleic acid-binding, OB-fold-like protein
	AT1G03820	unknown protein
	AT1G03830	guanylate-binding family protein
	AT1G18720	unknown protein
	AT1G18730	NDF6 NDH dependent flow 6
	AT1G18735	other RNA
	AT1G18740	unknown protein
	AT1G18745	NcRNA
	AT1G18750	AGL65 AGAMOUS-like 65
	AT2G23250	UGT84B2 UDP-glucosyl transferase 84B2
	AT2G23260	UGT84B1 UDP-glucosyl transferase 84B1
	AT2G23270	unknown protein
	AT2G23290	AtMYB70 myb domain protein 70
	AT5G44740	POLH
	AT5G44750	REV1
	AT5G44760	C2 domain-containing protein
	AT5G44770	Cysteine/Histidine-rich C1 domain family protein
