# Supplementary material for: Natural CMT2 Variation Is Associated With Genome-Wide Methylation Changes and Temperature Seasonality
Source: PLoS Genet. 2014 Dec 11;10(12):e1004842. doi: 10.1371/journal.pgen.1004842 (PMC4263395; doi:10.1371/journal.pgen.1004842)
Supplement: S3 Table — Experimental data of the heat-stress treatment on Col-0 and cmt2 knockouts. (PDF) [file pgen.1004842.s049.pdf]

Table S3: Experimental data for the heat-stress treatment of Col-0 and *cmt2-5* plants

experiment	accession	total	alive	dead
1	cmt2-5	28	17	11
1	cmt2-5	25	21	4
1	Col-0	25	12	13
1	Col-0	23	5	18
2	Col-0	28	8	20
2	Col-0	30	4	26
2	cmt2-5	26	10	16
2	Col-0	25	4	21
2	cmt2-5	19	11	8
2	Col-0	29	11	18
3	cmt2-5	25	20	5
3	Col-0	29	21	8
3	cmt2-5	26	16	10
3	Col-0	29	12	17
3	Col-0	29	13	16
3	Col-0	29	14	15
