# Supplementary material for: Natural CMT2 Variation Is Associated With Genome-Wide Methylation Changes and Temperature Seasonality
Source: PLoS Genet. 2014 Dec 11;10(12):e1004842. doi: 10.1371/journal.pgen.1004842 (PMC4263395; doi:10.1371/journal.pgen.1004842)
Supplement: S4 Table — Experimental data of root growth (mm) of Col-0 and cmt2 knockouts, with and without 6 h heat stress. (PDF) [file pgen.1004842.s050.pdf]

Col		cmt2	
control	6h heat	control	6h heat
11.839	13.48	17.375	20.214
10.911	0.364	28.15	22.313
20.818	22.539	26.546	23.846
8.357	0	20.006	22.25
13.656	1.214	19.581	18.644
23.621	21.834	8.576	9.141
16.425	18.095	23.061	18.037
26.866	7.785	26.788	3.843
7.801	14.861	23.047	9.019
23.397	8.013	33.445	24.753
11.484	0.243	27.716	6.607
8.684	10.881	21.39	33.157
18.94	10.902	23.379	19.084
21.055	1.093	14.33	5.901
21.355	0	19.115	22.115
26.758	16.586	29.795	1.75
25.958	6.19	18.046	1.996
23.438	0.777		6.376
23.419	12.242		17.121
22.792	0.384		15.948
21.526	1.341		7.113
26.55	0.738		19.502
16.929	0.619		6.085
29.122	24.244		14.354
27.851	25.941		1.573
24.558	10.512		16.381
24.275	0.243		5.892
30.535	23.916		
30.152			
18.197			
