# Supplementary material for: Natural CMT2 Variation Is Associated With Genome-Wide Methylation Changes and Temperature Seasonality
Source: PLoS Genet. 2014 Dec 11;10(12):e1004842. doi: 10.1371/journal.pgen.1004842 (PMC4263395; doi:10.1371/journal.pgen.1004842)
Supplement: S1 Text — Additional results, methods, source-code and comments. (PDF) [file pgen.1004842.s051.pdf]

Additional Results

CMT2 is a potential target for the nonsense-mediated RNA decay (NMD) pathway

The $CMT2_{STOP}$ allele is predicted to produce an mRNA with a pre-mature translation termination codon, which makes it a likely target for the nonsense-mediated mRNA decay (NMD) pathway (Nicholson and Mühlemann 2010). Thus, accessions carrying $CMT2_{STOP}$ may have lower transcript abundance than those with wild-type *CMT2*. To test whether this was the case, we evaluated the levels of *CMT2* mRNA using two publicly available datasets. First, we analyzed the 19 genomes project data (Gan et al. 2011) that contained full genome sequences and transcriptome for 19 *A. thaliana* accessions, two of which (Ct-1 and Kn-0) are part of the RegMap panel and carry the $CMT2_{STOP}$ mutation. The difference in mRNA abundance was highly significant between *CMT2* mutant and wild-type accessions ($P = 6.9 \times 10^{-5}$), with a higher expression in the wild type. A similar analysis was done using a larger data set from[7], with CMT2 genotypes called from whole-genome re-sequencing data and using RNA-seq data from leaf tissue in 14 $CMT2_{STOP}$ and 92 $CMT2_{WT}$ accessions. As in the other dataset, the average mRNA abundance was higher for the CMT2 wild-type accessions, but here the difference was not significant ($P = 0.14$). However, the mRNA levels were significantly higher among the four $CMT2_{STOP}$ accessions that displayed a methylation pattern resembling that of the $CMT2_{WT}$ in the analysis above (t-test; $P = 0.01$). When those lines were omitted, the average mRNA level was significantly higher in the $CMT2_{WT}$ accessions than in the ten remaining $CMT2_{STOP}$ accessions (t-test; $P = 0.02$). Together, these results indicate that *CMT2* mRNA levels are often lower in $CMT2_{STOP}$ than in *CMT2* wild-type accessions. This is consistent with the notion that the premature stop codon does interfere with translation as expected.

Methods

Plant Material

A T-DNA insertion line (SAIL_906_G03, *cmt2*-5) (Figure S45) was ordered from NASC and seeds were produced from confirmed homozygous insertion plants. *CMT2* (AT4G19020) transcripts spanning the insertion site were assayed by RT-PCR with the following primers: forward_CMT2 CTCATTACCCCCAGAGGAGAG, reverse_CMT2 AAAGTTTGGGTCGAGGAAGAG. As a control a *PP2A* gene (AT1G13320) was used with primers forward_PP2A ATTCCGATAGTCGACCAAGC, reverse_PP2A: AACATCAACATCTGGGTCTTCA. RNA was extracted from five seedlings using the Trizol method as described previously (Steinbach and Hennig 2014). RNA was DNA treated with DNase I (Thermo Scientific) according to the manufacturer's instructions. 1 μg of RNA was used to synthesize cDNA with the RervertAid First Strand cDNA synthesis Kit (Thermo scientific) according to manufacturer's instructions. No CMT2 transcripts spanning the insertion site were detected strongly suggesting that *cmt2*-5 is a null allele.

Source Code

Example R source code for calculating HEM genomic kinship matrix

Here, we use the example data in the **bigRR** package: http://cran.r-project.org/web/packages/bigRR/ to illustrate how an ordinary identity-by-state (IBS) kinship matrix can be update to a HEM genomic kinship matrix. The full theoretical details on this procedure are provided in[13].

```
# load the bigRR package
require(bigRR)

# load the example data
data(Arabidopsis)
X <- matrix(1, length(y), 1)
Z <- scale(Z)

# fitting SNP-BLUP, i.e. a ridge regression on all the markers across the genome
SNP.BLUP <- bigRR(y = y, X = X, Z = Z, family = binomial(link = 'logit'))

# calculate HEM (heteroscedastic effects model) genomic kinship matrix
w <- as.numeric(SNP.BLUP$u^2/(1 - SNP.BLUP$leverage))
wZt <- sqrt(w)*t(Z)
G <- crossprod(wZt)
```

Temperature seasonality phenotype preparation in R

We present the source code for phenotyping of temperature seasonality in Euro-Asia. The data downloaded from http://www.worldclim.org/ were processed using the following code to obtain an object readable by the **raster** package: http://cran.r-project.org/web/packages/raster/.

```
# load the original data files
bil_files <- grep(".bil", dir("tmean_30s_bil Folder/"), value = T)
bil_file_order <- as.numeric(sub(pattern = ".+_([0-9]+).bil",
                    x = bil_files, replacement = "\\1"))
bil_files <- bil_files[order(bil_file_order)]

# create rasters
WorldClim_stack <- stack()
for (bil_file in bil_files){
r <- raster(paste("tmean_30s_bil Folder/",bil_file, sep = ""))
WorldClim_stack <- addLayer(WorldClim_stack, r)
```

```
}

# temperature seasonality calculation
r_mean <- calc(WorldClim_stack, mean)
save(r_mean, file = "WorldClim_mean.Rdata")
writeRaster(r_mean, file = "WorldClim_mean.raster")
r_sd <- calc(WorldClim_stack, sd)
save(r_sd, file = "WorldClim_sd.Rdata")
writeRaster(r_sd, filename = "WorldClim_sd.raster")
r_mean_corr <- r_mean/10 + 273.15
save(r_mean_corr, file = "WorldClim_mean_corr.Rdata")
r_coeff_var <- 100*(r_sd/10)/r_mean_corr

# output to a raster object
writeRaster(r_coeff_var, file = "WorldClim_coeff_var.raster")

# phenotyping at given coordinates
# (LONGITUDE and LATITUDE already loaded)
require(raster)
world_temp_seas <- raster('WorldClim_coeff_var')
temp_seas <- raster::extract(world_bio5, cbind(LONGITUDE, LATITUDE))
```

Comments

Statistical properties of the vGWAS in relation to population stratification

Inherent properties of the variance heterogeneity test decreases risk of identifying locally adapted alleles
An important property of the variance heterogeneity GWAS analysis is that it is inherently more powerful in detecting loci where the minor allele is associated with a higher variance than the major allele. In practice, there is no power to detect low-variance minor alleles in a GWAS setting[12,30]. Hence, the method facilitates detection of alternative (*i.e.* minor) alleles associated with a broader range of the climate variables than the reference (*i.e.* major) alleles. The method is powerful in finding associations to minor alleles associated with a broader range of climate-variables than the reference. Such alleles will, by definition, be present across a large proportion of the global population and due to this be considerably less affected by population structure. In **Figure 3** or **Figure S15** (top panel), we illustrate this property for the inferred loci using the *CMT2* locus as an example. The MDS-plot visualizes the distribution of the $CMT2_{STOP}$ allele across the population structure present in the RegMap collection using the pairwise genome-wide relationship between the accessions based on the first two principle-components of the kinship matrix. The link between the geographic origin of the

accessions and kinship is visualized by coloring the dots for each accession based on geographic origin. As expected, accessions from nearby regions (*e.g.* UK, Scandinavia and mainland Europe) are more related. The $CMT2_{STOP}$ allele is, however, not heavily confounded with population-structure and is present in most major sub-groups of the population (albeit with a higher frequency in Asia - see **Figure S36**).

Mixed models based vGWAS analyses to account for population structure via modeling of genome-wide kinship We statistically deal with the strong correlations that exist between climate & population structure (*e.g.* along east-west/north-south clines) using a mixed-model based approach accounting for genomic kinship combined with genomic control. This approach has earlier been shown to control type I errors (*i.e.* genome-wide *P*-value inflation) in structured populations. The major challenge in analyzing this population is thus not the false-positive rate, as also standard GWAS analyses can be implemented in the same mixed-models framework, but rather to avoid unacceptably high type II errors (*i.e.* low power) for traits confounded with population-structure. Traditional GWAS analyses model alleles to have a linear relationship with climate, which in practice means that they mostly coincide with the population-structure along geographic clines. Hence, analyses will either be prone to identify false associations (when population-structure is not accounted for), or be under-powered (when accounting for population-structure). Although this is not explicitly discussed in the earlier reports based on this data, this is the primary reason for their lack of genome-wide significant associations to individual adaptive loci. As illustrated in **Figure 3**, the variance-heterogeneity test identifies loci present across population strata, where the signal therefore remain even after accounting for population structure via the mixed-model approach. The independence between the effect of the inferred locus and population structure can be evaluated statistically by fitting a linear mixed model where the genotype is regressed on the genomic kinship, where the heritability differs from 0 when confounding is present. For *CMT2* this estimate is zero, showing that the *CMT2* genotype is not confounded with population structure in this data.

On the power of vGWAS and GWAS analyses in highly structured populations There are several reasons for why a low overlap is expected between the results from traditional GWAS/selective-sweep analyses (as performed earlier) and the variance-heterogeneity GWAS (vGWAS) used here. First, in the absence of population stratification, the GWAS is more powerful than the vGWAS. In the presence of population stratification, however, loci affecting the mean phenotype will often be highly confounded with population-structure as they are a main genetic mechanism leading to local adaptation. In order to infer such loci when controlling for population structure, the same alleles need to have been under selection in multiple, unrelated populations, which is apparently a rare event as no such loci could be detected in the earlier studies of climate adaptation. The population genetics forces acting on variance-controlling loci are still poorly explored. Studies have, however, shown that high- and low-variance alleles are likely to co-exist in the population over extended periods of time at a frequency balanced depending on fluctuations in the surrounding environment that the population adapts to[11]. Due to this, both alleles are more likely to be present across different population strata than mean affecting alleles and therefore be less confounded with population structure. In **Figure S36**, we exemplify this by visualizing the allele-frequency of the $CMT2_{STOP}$ allele across the major sub-populations in the RegMap

and 1001-genomes data. Although the uneven sampling across the regions in the 1001-genomes makes allele-frequency estimates uncertain, the overall picture shows that minor allele is present across all sub-populations at a lower frequency and that it has increased in frequency in Asia.

Second, a traditional GWAS searches for difference in means between genotypes, whereas the vGWAS searches for differences in variances between genotypes. As these are two different statistical properties of the phenotypic distribution, the basic assumption is that they are both statistically and biologically unrelated and consequently the loci identified by the two methods are not expected to overlap. Although some degree of overlap might be expected, *e.g.* in situations where the variance scales with the mean, the high-significance required to reach genome-wide significance in the testing, in practice only loci with strong, pure effects on one of the statistical moments seem to be able to reach such significance levels[12]. Formal comparisons between the results in the association studies will thus be misleading to the readers, as these will indicate that an overlap is to be expected. Results from evaluations of the overlap for sub-GWAS signals to explore the potential overlap of loci with weaker effects on both the mean and the variance shows some overlap (**Figure S40-S44**). It should be noted, however, that comparisons of overlap at individual loci is not appropriate at these significance levels due to the lack of proper control of the type I error rate. The overall conclusions from these comparisons is i) that the power is generally very low for the GWAS after control for population stratification and ii) that even the sub-GWAS overlap is low for the two methods, but the overlap that exists is consistent with the correlation between the climate variables.

Third, the earlier studies have also inferred loci using traditional selective-sweep mapping. These analyses are designed to infer hard selective-sweeps where (potentially) adaptive alleles are assumed to have increased in frequency due to directional selection. As discussed above, the population genomic dynamics of plastic alleles does not follow the same pattern as for alleles affecting the mean[11], leading to a co-existence of the alleles over prolonged periods of time. This means that they will not be surrounded by a traditional genomic footprint of directional selection that can be detected in a selective-sweep analysis and one would not expect any overlap between the loci inferred in the selective sweep and vGWAS based analyses.

References

X. Gan, O. Stegle, J. Behr, J. G. Steffen, P. Drewe, K. L. Hildebrand, R. Lyngsoe, S. J. Schultheiss, E. J. Osborne, V. T. Sreedharan, A. Kahles, R. Bohnert, G. Jean, P. Derwent, P. Kersey, E. J. Belfield, N. P. Harberd, E. Kemen, C. Toomajian, P. X. Kover, R. M. Clark, G. Rätsch, and R. Mott. Multiple reference genomes and transcriptomes for Arabidopsis thaliana. *Nature*, 477(7365): 419–423, Sept. 2011. doi: 10.1038/nature10414. URL http://eutils.ncbi.nlm.nih.gov/entrez/eutils/elink.fcgi?dbfrom=pubmed&id=21874022&retmode=ref&cmd=prlinks.

P. Nicholson and O. Mühlemann. Cutting the nonsense: the degradation of PTC-containing mRNAs. *Biochem Soc Trans*, 38(6):1615–1620, 2010.

Y. Steinbach and L. Hennig. Arabidopsis MSI1 functions in photoperiodic flowering time control. *Front Plant Sci*, 5(77), 2014.
